# Phenotypic screen of sixty-eight colorectal cancer cell lines identifies CEACAM6 and CEACAM5 as markers of acid resistance

**DOI:** 10.1073/pnas.2319055121

**Published:** 2024-03-19

**Authors:** Johanna Michl, Bobby White, Stefania Monterisi, Walter F. Bodmer, Pawel Swietach

**Affiliations:** ^a^Department of Physiology, Anatomy and Genetics, University of Oxford, Oxford OX1 3PT, United Kingdom; ^b^Department of Oncology, University of Oxford, Oxford OX3 7DQ, United Kingdom

**Keywords:** tumor acidity, acidosis, microenvironment, acid–base, metabolism

## Abstract

Elevated cancer metabolism releases lactic acid and CO_2_ into the under-perfused tumor microenvironment, resulting in extracellular acidosis. The surviving cancer cells must adapt to this selection pressure; thus, targeting tumor acidosis is a rational therapeutic strategy to manage tumor growth. However, none of the major approved treatments are based explicitly on disrupting acid handling, signaling, or adaptations, possibly because the distinction between acid-sensitive and acid-resistant phenotypes is not clear. Here, we report pH-related phenotypes of sixty-eight colorectal cancer (CRC) cell lines by measuring i) extracellular acidification as a readout of acid production by fermentative metabolism and ii) growth of cell biomass over a range of extracellular pH (pHe) levels as a measure of the acid sensitivity of proliferation. Based on these measurements, CRC cell lines were grouped along two dimensions as “acid-sensitive”/“acid-resistant” versus “low metabolic acid production”/“high metabolic acid production.” Strikingly, acid resistance was associated with the expression of *CEACAM6* and *CEACAM5* genes coding for two related cell-adhesion molecules, and among pH-regulating genes, of *CA12*. CEACAM5/6 protein levels were strongly induced by acidity, with a further induction under hypoxia in a subset of CRC lines. Lack of CEACAM6 (but not of CEACAM5) reduced cell growth and their ability to differentiate. Finally, CEACAM6 levels were strongly increased in human colorectal cancers from stage II and III patients, compared to matched samples from adjacent normal tissues. Thus, CEACAM6 is a marker of acid-resistant clones in colorectal cancer and a potential motif for targeting therapies to acidic regions within the tumors.

## Significance

The fluid-filled spaces between cancer cells can become substantially acidic in a tumor. This produces a harsh chemical microenvironment that forces cells to adapt or die. The surviving cells are likely to have aggressive features, so eliminating these populations is therapeutically desirable. By screening sixty-eight colorectal cancer lines, we grouped cells by how much acid they produce and how sensitive their growth is to acidity. We reasoned that acid-resistant phenotypes are better adapted for surviving the tumor microenvironment. Acid-resistant cell lines were characterized by high levels of CEACAM6, a protein found at the surface of cancer cells and also present in late-stage disease in human cancers. CEACAM6 may offer a means of improving drug delivery specifically to acidic tumor regions.

In solid tumors, the elevated metabolic activity of cancer cells and aberrant blood perfusion (1) lead to a substantial buildup of lactic acid and CO_2_, which lowers extracellular pH (pHe) of the tumor microenvironment (2–4). Low pHe normally reduces intracellular pH (pHi), but cancer cells are able to restore physiological acid–base balance through adaptations involving pHi-regulatory proteins (5). Changes in acid–base chemistry can influence cellular functions (1, 6) and are considered both a consequence of and contributor to cancer progression. Notably, protonation is a powerful posttranslational modification (7, 8) that can influence the outcomes of certain cancer-promoting mutations, for example, *EGFR* (9). Additionally, tumor acidosis leads to metabolic programming of cancer cells (10–12), inhibits T cell function (13, 14), influences metastatic potential (15–17), and compromises the efficacy of certain therapies (18).

Aberrant perfusion also predisposes tumors to becoming hypoxic, which has long been considered a major microenvironmental factor in shaping cancer progression (19). Acidity is oftentimes a consequence of hypoxia because activation of hypoxia-inducible factor (HIF) causes a metabolic shift toward lactic acid fermentation. However, acidity and hypoxia should be considered distinct environmental variables because of their different sources and biological actions. There are instances where fermentative metabolism is stimulated even in the presence of oxygen (Warburg effect), which means that hypoxia and acidity are not stoichiometrically coupled. For example, acidosis has been shown to occur at the normoxic tumor/stroma interface, whereas hypoxic conditions are more likely at the tumor core (20). Consequently, cancer cells in tumors can be exposed to various combinations of acidic and hypoxic stress.

Unlike hypoxia, for which adaptive and survival mechanisms have been researched extensively (21–23), the cellular responses to acidosis are less clear. This is partly due to the challenges associated with controlling pH in cell culture (24). Furthermore, the effects of pH are transmitted across multiple signaling pathways, rather than one dominant mechanism, like the case of HIF in hypoxia. The notion that acidosis is not merely a passive end- product of metabolism but a bona fide signal is supported by findings that acidosis can change the expression of genes (20, 25) independently of hypoxia (25, 26). Surviving under acidosis is also likely to require bespoke adaptive responses; for example, low pH blocks fermentative metabolism, which forces cells to rely on oxidative phosphorylation (OXPHOS). We found that cancer cell survival at low pHe requires *NDUFS1* and other OXPHOS genes (27), whereas the same genes are dispensable for cell growth under hypoxic conditions (23). We also described an adaptation to tumor acidosis that involves the degradation of the acid-loading transporter anion exchanger 2 (AE2) (5), thereby restoring pHi to a range that is more favorable for tumor progression and invasiveness (28). Others have shown that long-term exposure to acidity drives glutamine metabolism and OXPHOS (29) which are crucial for survival under acidosis, but not hypoxia (27). The case for disarming these acid-driven adaptations therapeautically is compelling but this would require a targeted approach that delivers agents to acid-adapted cells, with minimal actions in normal tissues.

A limitation of many discovery pipelines is that they do not use a sufficiently comprehensive range of cell lines to cover the genetic heterogeneity in human cancers. For example, large-scale experiments such as CRISPR/Cas9 screens are feasible on a few cell lines at best (27). Consequently, key characteristics of acid-resistant cells may have evaded discovery. Understanding the phenotypes that survive under acidosis necessitates analysis of a large panel of cell lines to give adequate power for detecting acid-resistant traits. To provide therapeutically valuable insight, these cell lines should be representative of the range of (epi)genetic variation found in the cancer under investigation (30).

To gain insight into the acid-resistant phenotype, we tested a panel of 68 colorectal cancer (CRC) cell lines, representing various combinations of mutations. We reasoned that the minimum information needed to classify pH-related phenotype is to determine the cell line’s fermentative rate, a key source of acidity, and the pH sensitivity of cell growth. We correlated acid resistance with gene expression data and identified several correlating genes. Of these, CEACAM5 and CEACAM6 are attractive markers because they are carcinoembryonic antigen-related cell adhesion molecules (CEACAMs), a family of immunoglobulin-related glycoproteins (31–33). CEACAMs are involved in cell–cell recognition and regulate tissue architecture and neovascularization, T cell proliferation, and insulin homeostasis. As receptors for host-specific viruses and bacteria, CEACAMs are implicated in mechanisms of pathogen–host coevolution. CEACAM5, the original carcinoembryonic antigen (CEA), has long been considered a promising avenue for targeted therapy of colorectal cancer due to its overexpression in many tumors (34). Indeed, a test for serum CEA has been in clinical use since the 1960s to indicate and track CRC recurrence (35, 36). More recently, CEACAM6 has been noted for being an independent prognostic factor of CRC (37). We found that both CEACAM5 and CEACAM6 are strongly induced by acidity, with a further induction under hypoxia. We propose that a suitable drug delivery system, which targets CEACAM6 or CEACAM5 as markers of acid-resistant cancer cells, could be a promising molecularly stratified therapeutic strategy for CRC.

## Results

### Measuring the pH-related Phenotype in a Panel of 68 Colorectal Cancer Cell Lines.

**Fig. 1.**
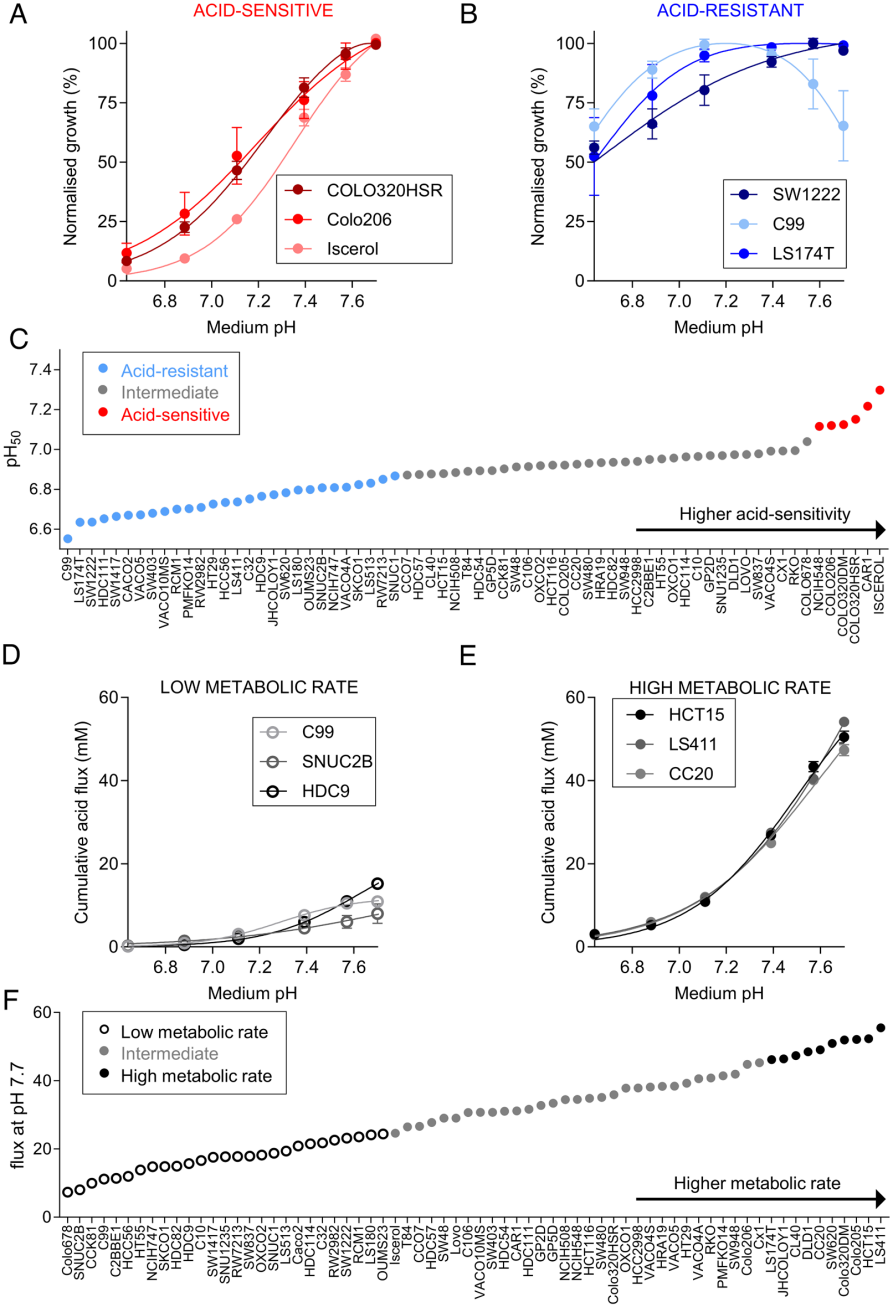
Effect of medium pH on CRC cell growth and metabolic acid production. (*A* and *B*) Peak-normalized growth in biomass [sulforhodamine B (SRB) absorbance] after 6 d of culture from a range of starting pHe. Data are shown for representative acid-sensitive (COLO320HSR, Colo206, Iscerol) and acid-resistant (SW1222, C99, LS174T) cell lines. SRB absorbance values were normalized to growth at interpolated optimum pHe. Mean ± SEM of 2 to 5 independent repeats (triplicate technical replicates). (*C*) Ranking of cell lines according to pH_50_ value (pHe at which growth is halved relative to optimal growth). Using Gaussian mixture modeling, cell lines were grouped into acid-sensitive, intermediate, or acid-resistant. (*D* and *E*) Cumulative acid production at pHe 7.4 based on medium acidification measurements (phenol red absorbance) and buffering capacity (assuming open system for CO_2_) in cell lines representative of low metabolic rate (C99, CCK81, and HDC9) and high metabolic rate (LS411, DLD1, and CC20). (F) Ranking of cell lines according to metabolic flux at pHe 7.7. Cell lines were classified as “high metabolic rate”, “control” and “low metabolic rate.”

**Fig. 2.**
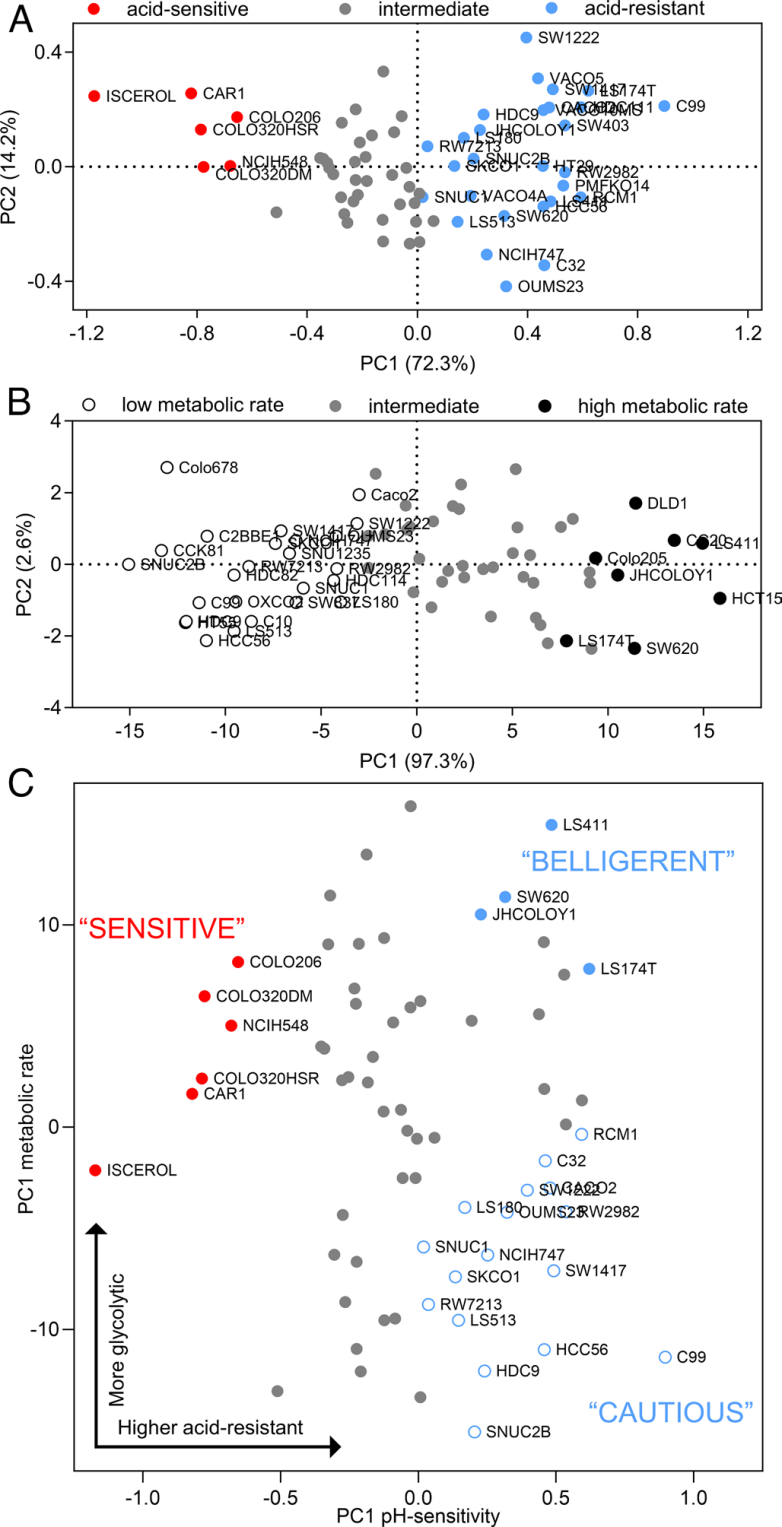
Grouping of cell lines according to acid sensitivity and metabolic acid production. (*A* and *B*) Principal component analysis of cell lines based on their normalized cell growth or cumulative acid production at six pHe values. (*C*) Acid resistance (PC1 of Fig. 2*A*) vs. metabolic acid production (PC1 of Fig. 2*B*) for the panel of 68 CRCs.

We performed assays on 68 CRC lines that include replication error (RER)-positive and RER-negative lines and carry various combinations of mutations (*SI Appendix*, Table S1). To measure cell growth, we quantified biomass by the sulforhodamine B (SRB) assay after six days of culture over a range of starting pHe. Initial medium pH was adjusted by varying [HCO_3_^–^] under an atmosphere of 5% CO_2_. pH sensitivity of growth was largely independent of seeding density for selected cell lines (*SI Appendix*, Fig. S1 *A*–*D*) and was therefore standardized to 4,000 cells/well in a 96-well plate for screening the entire CRC cell line panel. Fig. 1 *A* and *B* shows exemplar cell growth data from cell lines determined to be acid-sensitive (COLO320HSR, Colo206, and Iscerol) and acid-resistant (SW1222, C99, and LS174T), normalized to the interpolated optimal pHe. Acid-sensitive cell lines showed strongly reduced growth (<20% of maximum) at pHe 6.6, whereas acid-resistant cell lines were able to grow relatively well (>50% of maximum) at pHe 6.6. The survival curves can be summarized by a pH_50_ value that determines the pHe at which growth is halved, relative to optimal growth (Fig. 1*C*). We also measured medium acidification using Phenol Red absorbance ratiometrically over 6 d. By time integration, this determined the cumulative acid production from a starting pHe of 7.7. Fig. 1 *D* and *E* shows examples of cell lines with low (C99, CCK81, and HDC9) and high (LS411, DLD1, and CC20) metabolic acid production, reflecting fermentative rate. Cell lines were ranked according to their metabolic flux (Fig. 1*F*). Based on Gaussian mixture modeling (GMM) (38) of pH_50_ and metabolic flux data, cell lines were grouped into categories of “acid-sensitive,” “intermediate” or “acid-resistant,” as well as “low metabolic rate,” “intermediate” and “high metabolic rate” (*SI Appendix*, Fig. S2 *A* and *B*). We also performed principal component analysis (PCA) using the shape of the pHe-sensitivity curves of growth as input (Fig. 2*A*). A similar analysis was performed for metabolic acid production (Fig. 2*B*). By plotting the magnitude of the first principal component (PC1) of acid sensitivity against PC1 for metabolic acid production, we assigned cell lines to distinct phenotypic groups, from which exemplar cells could be selected for further investigation (Fig. 2*C*). Acid-resistant lines were described as “belligerent” if they also had a high acid production rate or “cautious” if their acid production rate was low. Acid-sensitive lines with a high metabolic rate were described as “sensitive.” Intriguingly, no lines fell in the category of “acid-sensitive with low metabolic rate,” which could be described as “vulnerable.”

### Validation of pH Phenotypes Using Competition Assays.

**Fig. 3.**
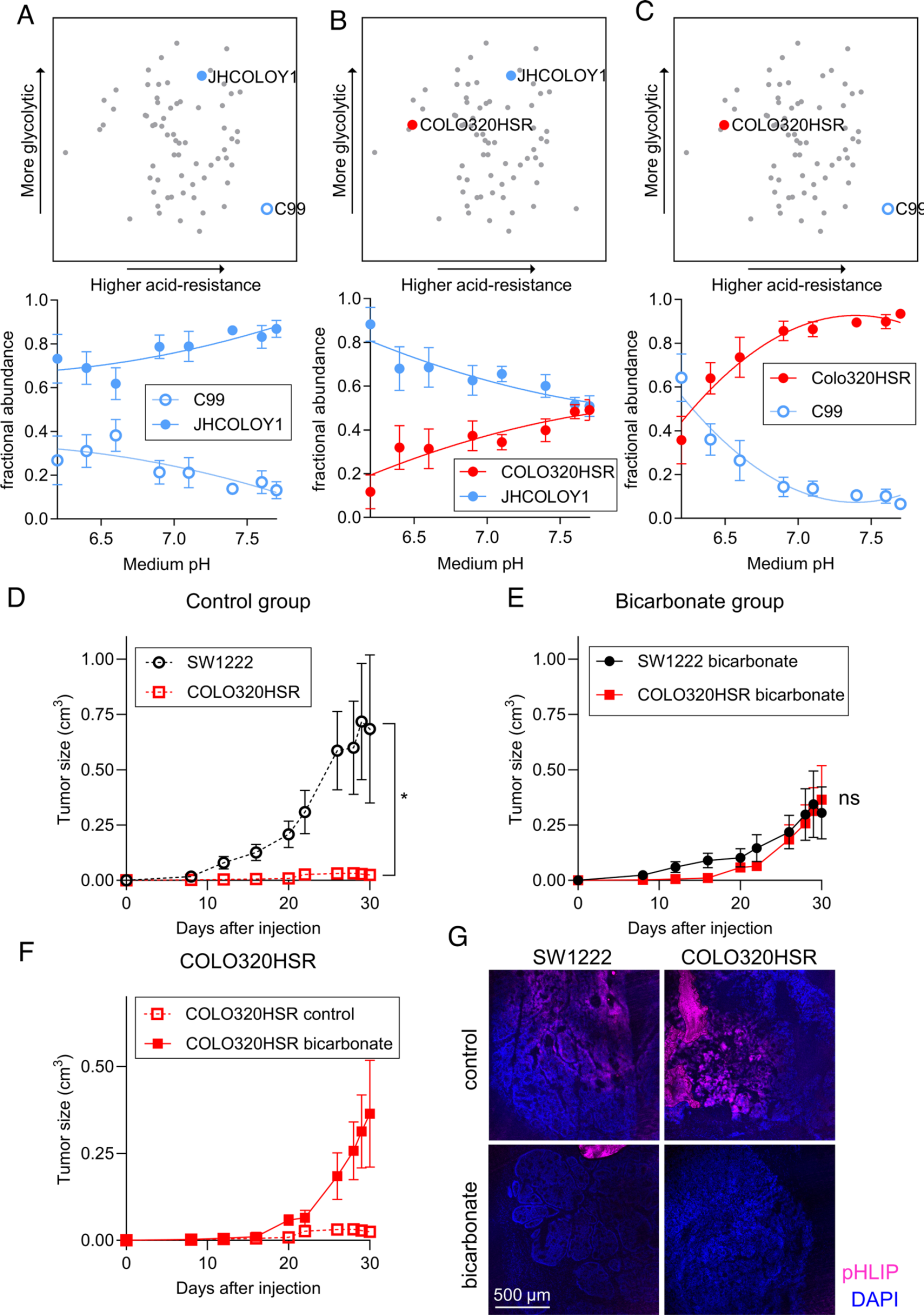
pH-related phenotype is maintained in 2D competition assays and in vivo xenograft experiments. (*A*–*C*) Results of 2D coculture experiments between a pair of CRC lines with distinct pH-related phenotypes, as indicated in the PCA plots. Cell lines were eGFP- or mCherry-labeled to distinguish growth and seeded in a 1:1 ratio. Culture was performed in media of a range of pHe for 8 d. Fractional abundance was determined by measuring the area covered by eGFP or mCherry fluorescence using high-throughput imaging. Coculture experiments included combinations of representative cell lines of “acid-sensitive” (COLO320HSR), “acid-resistant with low metabolic rate” (C99), and “acid-resistant with high metabolic rate” (JHCOLOY1) cell lines. Mean ± SEM of four independent repeats (triplicate technical replicates). (*D*) SW1222 (“acid-resistant” cell line) and COLO320HSR (“acid-sensitive” cell line) tumor volume in mice receiving regular drinking water. These cell lines have matching metabolic acid production rates in vitro. Data represent paired measurements from mice injected with SW1222 cells in their left flank and COLO320HSR cells in their right flank (mean ± SEM of six animals). (*E*) SW1222 and COLO320HSR tumor volume in mice receiving oral bicarbonate treatment. Data represent paired measurements from mice injected with SW1222 cells in their left flank and COLO320HSR cells in their right flank (mean ± SEM of six animals). (*F*) Comparison of tumor volume of COLO320HSR cells in control and sodium bicarbonate–treated animals (mean ± SEM of six animals). (*G*) Representative images of Cy5.5-conjugated pH-(low)-insertion peptide (pHLIP, red) and Hoechst-33342 (blue) in the fresh frozen tumor sections of SW1222 and COLO320HSR xenografts in animals receiving oral bicarbonate or water (control). (Scale bar, 500 μm.)

We predicted that cells with contrasting pH-related phenotypes may have distinct growth trajectories when cultured together over a range of starting pHe. To test this, we performed competition assays between cell lines of contrasting pH-related classification (Fig. 2*C*), transfected with spectrally resolvable fluorescent proteins (eGFP and mCherry). First, we performed a 1:1 coculture between two acid-resistant cell lines that differed in metabolic rate: high in JHCOLOY1 and low in C99. At the end of 8 d of culture in media over the pHe range 6.2 to 7.7, we measured the relative abundance of area covered by eGFP or mCherry-fluorescence using high-throughput imaging. JHCOLOY1 outcompeted C99, regardless of pHe, which can be explained by their higher metabolic rate (Fig. 3*A*). Next, we cocultured COLO320HSR cells (acid-sensitive with intermediate metabolic rate) with JHCOLOY1 cells (acid-resistant with high metabolic rate) in a 1:1 seeding ratio. The hypothesis here was that acid resistance confers a survival advantage that manifests at low pHe. Indeed, both cell lines showed similar growth under alkaline conditions, but JHCOLOY1 cells became more abundant at low pHe (Fig. 3*B*). Last, we compared COLO320HSR cells with C99 cells (Fig. 3*C*). This combination is less intuitive to predict because COLO320HSR has the advantage of a higher metabolic rate whereas C99 is more acid-resistant. Strikingly, COLO320HSR cells had a growth advantage over a wide pHe range, indicating the importance of metabolic rate in promoting growth. However, under profoundly acidic conditions (pHe < 6.3), C99 cells were able to outcompete COLO320HSR cells. Under acidic conditions, acid sensitivity of growth can overcome the advantage of a raised metabolic rate. These results indicate that pH-related phenotypes are stable and can influence cell growth in line with predictions borne from their pH-related characteristics.

### Xenografts of Acid-sensitive Cells Grow Faster with Oral Bicarbonate Therapy.

Building on in vitro results, we investigated whether pH-related phenotypes are also retained in vivo. For this, we injected acid-resistant SW1222 and acid-sensitive COLO320HSR cells subcutaneously to the left and right flanks, respectively, of immunodeficient nude mice to establish paired xenografts. In the cohort of 12 mice, half were given sodium bicarbonate (400 mM) in drinking water, and control animals had access to regular drinking water. Others have shown previously that oral bicarbonate raises tumor pHe in mice by systemic buffer loading (39). In the control (water) group, COLO320HSR xenografts showed very slow growth, compared to SW1222 xenografts (Fig. 3*D*). This result indicates that when tumors undergo acidification, acid-resistant cells (SW1222) have a substantial growth advantage. Conversely, in the bicarbonate group, growth of COLO320HSR xenografts was strongly stimulated, reaching levels similar to SW1222 xenografts (Fig. 3*E*). Therefore, bicarbonate treatment was able to rescue the growth of COLO320HSR xenografts (i.e., acid-sensitive cells), ostensibly by buffering the growth-hindering effect of tumor acidosis (Fig. 3*F*). The alkalinizing effect of oral bicarbonate on tumor pHe was confirmed by imaging Cy5.5-conjugated pH-low insertion protein (pHLIP) injected to mice as a terminal procedure. A strong pHLIP signal (acidic regions) was detected in xenografts of control animals. pHLIP signal was reduced in tumors from animals of the bicarbonate treatment group (Fig. 3*G*).

### Correlating pH-related Phenotype with Gene Expression and Driver Mutations.

**Fig. 4.**
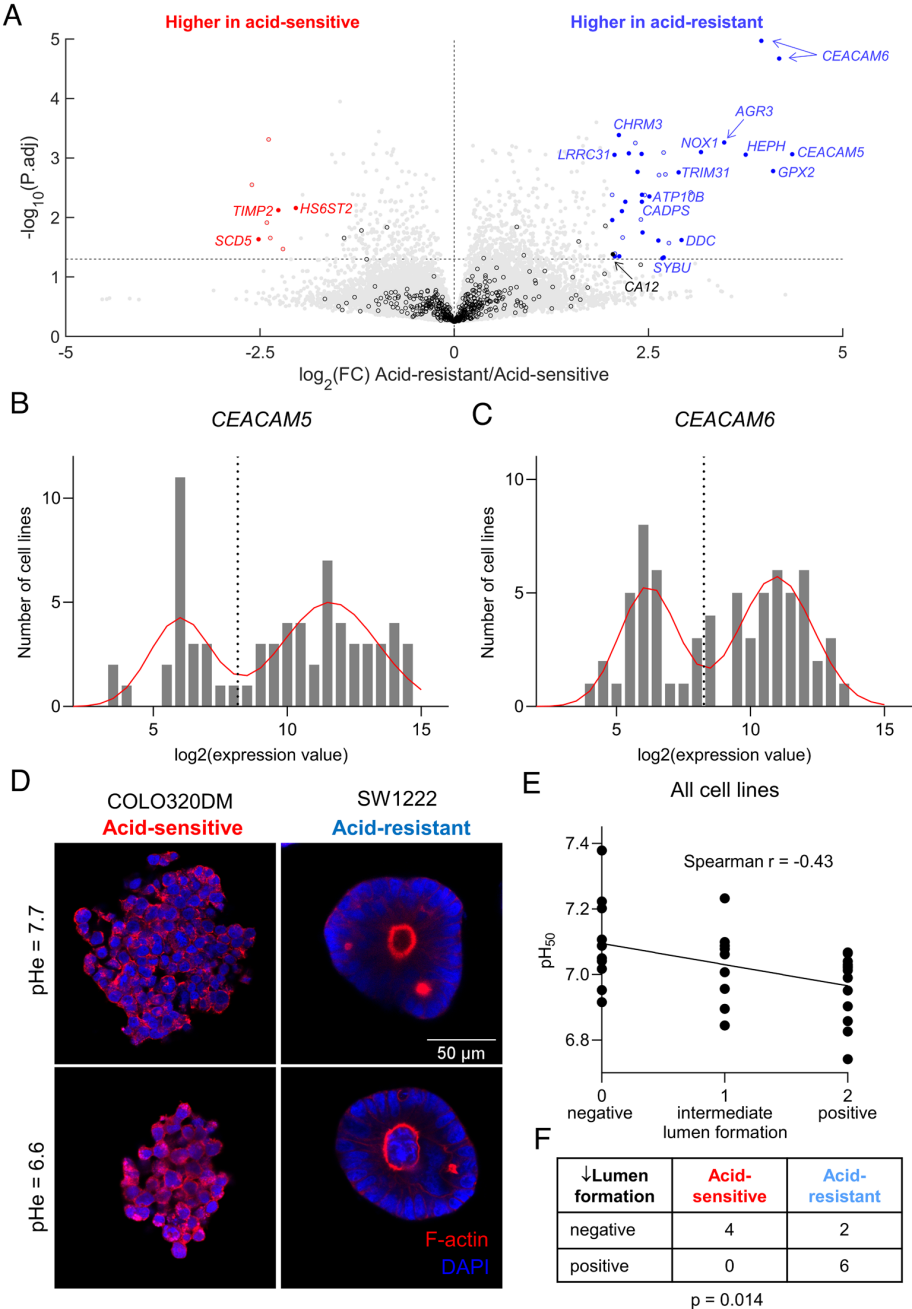
Correlation between pH-related phenotype and mRNA expression or differentiation status. (*A*) Volcano plot highlighting genes that are differentially expressed between cell lines classified as “acid-sensitive” (six cell lines) or “acid-resistant” (28 cell lines), as indicated in Fig. 1*F*. Note that mRNA data for some genes include multiple probe sets. Empty symbols denote genes that are not bimodally distributed. Filled symbols, labelled with gene name, denote genes that are bimodally distributed. Black symbols denote genes related to pH regulation. Red and blue symbols denote other genes that are up-regulated in acid-sensitive lines and up-regulated in acid-resistant lines, respectively. Statistical testing was done by multiple unpaired *t* tests with multiple comparisons correction. The horizontal line denotes *P* = 0.05. (*B* and *C*) mRNA expression of CEACAM5 and CEACAM6 within a panel of 68 colorectal cancer cell lines. Expression levels based on microarray analysis are given as log_2_ on the *x* axis, and numbers of samples with given expression levels on the *y* axis. The continuous curves are fitted mixed normal distributions, and the vertical dotted red line marks the best estimate for separating low and high levels of expression using Gaussian mixture modeling (GMM). (*D*) Images of single-cell colonies grown in Matrigel of cell lines representative of “acid-sensitive” (COLO320DM) or “acid-resistant” cell lines (SW1222). Colonies were stained with DAPI and TRITC-phalloidin (for F-actin labeling). Cells were cultured in a layer of Matrigel covered with medium of either pHe 7.7 or pHe 6.6 for 10 to 14 d. Images are representative of two independent repeats, carried out in technical duplicate. (*E*) Correlation between lumen formation phenotype [“negative” (score=0), “intermediate” (score=1), or “positive (score=2) and pH_50_ value (pH_50_ value represents the pHe at which growth is halved relative to that at the optimum pHe]. Spearman regression analysis shows a negative correlation between lumen differentiation status and pH_50_ values. Lumen formation data are representative of two independent repeats carried out in technical duplicate. (*F*) The contingency table shows a correlation between positive lumen formation and a “acid-resistant” phenotype (Chi-square test).

Correlating phenotype with gene expression is an unbiased approach for identifying novel genes linked to resistance to extracellular acidity. We performed a statistical analysis (multiple comparisons–corrected *t* tests) of differentially expressed genes (DEGs) for six ‘‘acid-sensitive” vs. twenty-eight ‘‘acid-resistant’’ cell lines identified by the phenotypic classification shown in Fig. 1*E*. The analysis shown in Fig. 4*A* identified a higher expression of the surface-expressed glycoprotein CEACAM5 (previously called CEA) and its close relative CEACAM6 among acid-resistant cell lines. Neither CEACAM5 nor CEACAM6 have previously been linked to resistance to tumor acidity. Among genes previously linked to pH, *CA12* was more highly expressed in acid-resistant cell lines. Using GMM, we showed that *CEACAM5* and *CEACAM6* mRNA levels in the panel of 68 colorectal cancer cell lines were bimodally distributed (Fig. 4 *B* and *C*). We found a strong correlation between cell lines with high *CEACAM5* or *CEACAM6* expression and their acid-sensitivity phenotypes by Chi-square analysis (*SI Appendix*, Fig. S2 *C* and *D*). Next, we tested for a correlation between acid sensitivity and the genetic background of the cell lines (*SI Appendix*, Table S2). This identified a positive correlation between mutations in *KRAS* and acid resistance (*P* = 0.026). However, acid resistance was not linked to other known driver mutations, nor RER- or epithelial mesenchymal transition (EMT)- status.

### Correlating pH-related Phenotype with Stem Cell Differentiation and Lumen Formation.

Single cancer stem cells (CSC) can be identified from their ability to produce large, lumen-containing colonies in 3D Matrigel cultures (40). We assessed the ability of a subgroup of 34 CRC cell lines, including acid-resistant, intermediate, and acid-sensitive, to form lumens as a read-out for their ability to differentiate. An example of lumens formed by an acid-resistant cell line (SW1222) and lack of lumen formation in an acid-sensitive cell line (COLO320DM) is shown in Fig. 4*E*. Moreover, lumen formation in SW1222 cells persisted at low pHe. A gallery of F-actin-labeled colonies from different cell lines is shown in *SI Appendix*, Fig. S3. Interestingly, several acid-resistant cell lines (e.g., HCC56, LS174T) were also able to form lumens. However, several cell lines characterized by typical lumen structures (HT55 and T84) showed only intermediate pH sensitivity. Conversely, acid-sensitive cell lines, such as COLO320HSR, Colo206, or Iscerol, were unable to form luminal structures (*SI Appendix*, Fig. S3). CEACAM5 was previously found almost exclusively in the lumens of SW1222 cells (40), suggesting a link between acid resistance and the ability of the cell lines to differentiate. Indeed, we observed a correlation between acid resistance (pH_50_) and lumen formation score (Fig. 4 *F* and *G*), suggesting that factors promoting differentiation also favor acid resistance.

### *CEACAM6* and *CEACAM5* Expression Is Induced by Acidity.

**Fig. 5.**
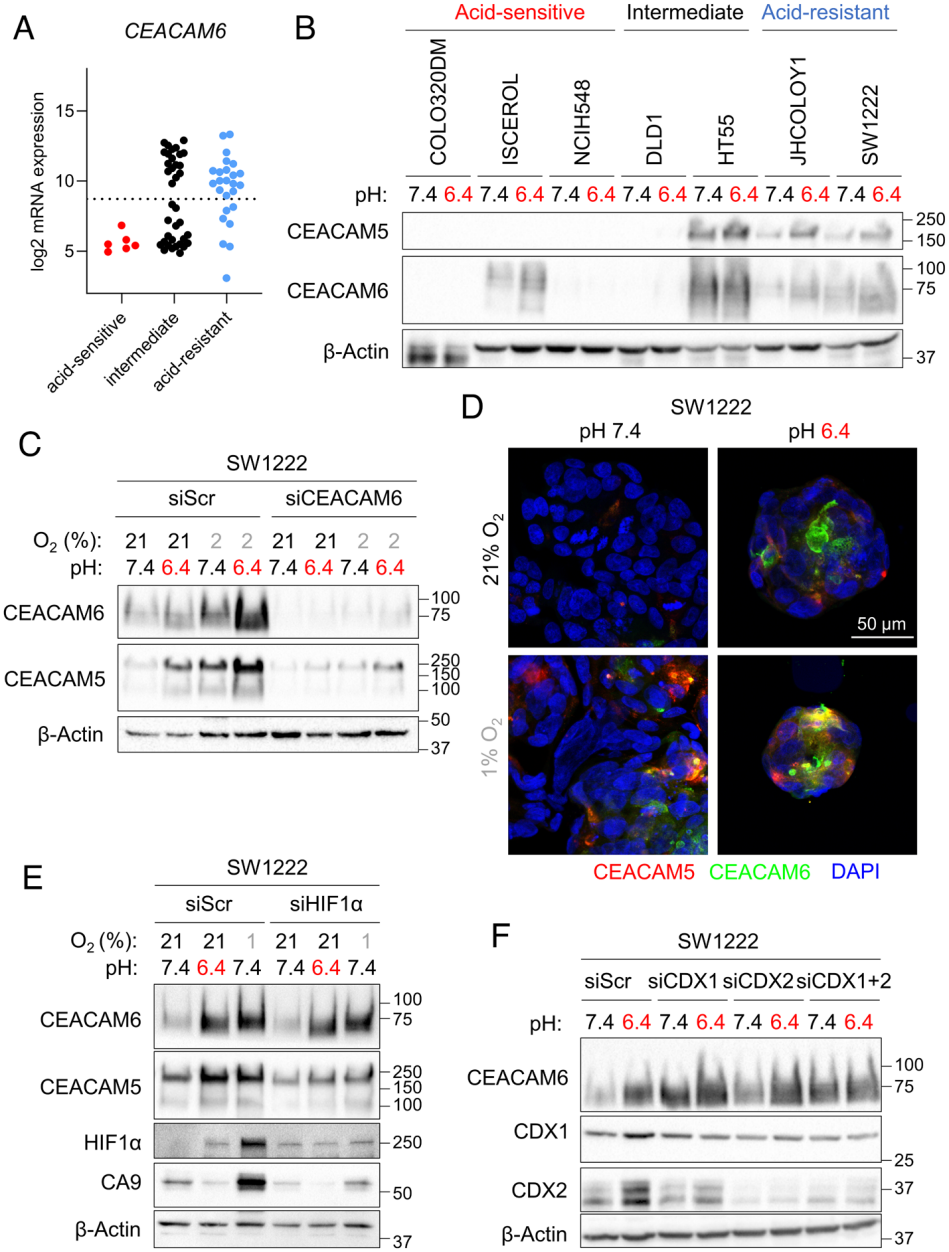
CEACAM5 and CEACAM6 are induced by acidity and hypoxia. (*A*) mRNA expression levels of *CEACAM6* in “acid-sensitive”, “intermediate” and “acid-resistant” cell lines obtained by microarray analysis. The dotted line represents the cutoff between low and high mRNA expression determined by GMM. (*B*) Western blot of lysates from COLO320DM, ISCEROL, NCIH548, DLD1, HT55, JHCOLOY1, and SW1222 cells treated for 72 h with media of pHe 7.4 or pHe 6.4. (*C*) Western blot of lysates from SW1222 cells transfected with siScr or siCEACAM6, followed by treatment for 72 h with media of pHe 7.4 or pHe 6.4 under 21 or 2% O_2_. (*D*) Immunofluorescence staining for CEACAM6 (green) and CEACAM5 (red) in SW1222 cells treated with media of pH 7.4 or pH 6.4 for 72 h under 21 or 2% O_2_ conditions. Images are representative of three independent repeats. (*E*) Western blot of lysates from SW1222 cells transfected with siScr or siHIF1α, followed by treatment for 72 h with media of pHe 7.4 or pHe 6.4 under 21 or 1% O_2_ conditions. (*F*) Western blot of lysates from SW1222 cells transfected with siScr, siCDX1, siCDX2 or siCDX1+2, followed by treatment for 48 h with media of pHe 7.4 or pHe 6.4.

Most acid-resistant cell lines had a significantly higher expression of *CEACAM6* mRNA compared to acid-sensitive cell lines (Fig. 5*A*). Although we also found enrichment of CEACAM5, we focused further studies on CEACAM6 as it was more strongly correlated with acid resistance and had been less extensively explored as a target for cancer therapy. The association between acid resistance and *CEACAM6* expression could be linked to the proteins’ role in cell differentiation since cell lines with high *CEACAM5/6* expression are more likely luminal-forming (*SI Appendix*, Table S3). We tested the effect of extracellular acidity (pHe 6.4) on CEACAM5 and CEACAM6 protein levels on a panel of seven cell lines, ranging from acid-sensitive to acid-resistant phenotypes (Fig. 5*B*). As expected, both CEACAM5 and CEACAM6 expression was higher in acid-resistant cell lines. Interestingly, the acid-sensitive line Iscerol expressed CEACAM6, but CEACAM5 was absent. Importantly, upon treatment with acidic medium (pHe 6.4) for 72 h, CEACAM5 and CEACAM6 abundance increased in the majority of positive cell lines. We confirmed that acidic medium induced *CEACAM6* mRNA levels in SW1222, C99, and JHCOLOY1 cells (*SI Appendix*, Fig. S4 *A*–*C*). In cell lines that were negative for CEACAM6 at pHe 7.4 (e.g., COLO320DM), treatment with acidic medium was not able to induce CEACAM6 protein (*SI Appendix*, Fig. S4*E*). This suggests that CEACAM6 is a marker of acid-resistant cells that becomes more abundant under acid stress; in contrast, acid-sensitive lines retain low expression irrespective of pHe.

We next tested whether hypoxia could influence protein levels of CEACAM5 or CEACAM6. As previously reported in other cell lines (41), CEACAM5 levels in SW1222 cells were increased after 72h of culture under hypoxic conditions (Fig. 5*C* and *SI Appendix*, Fig. S4*D*). When cells were exposed to a combination of hypoxia and acidity (pHe 6.4), we observed a further increase of both CEACAM5 and CEACAM6 levels in the acid-resistant cell lines SW1222 (Fig. 5*C* and *SI Appendix*, Fig. S4*D*) and C99 (*SI Appendix*, Fig. S5*B*). However, in acid-resistant JHCOLOY1 cells, CEACAM5/6 were induced by acidity, but not hypoxia (*SI Appendix*, Fig. S5*C*). CEACAM5/6 induction did not occur in the acid-sensitive cell line COLO320DM (*SI Appendix*, Fig. S4*E*), and only mildly in HT55 cells, which were categorized as intermediate acid sensitivity (*SI Appendix*, Fig. S5*A*). When CEACAM6 expression was reduced using siRNA-mediated knockdown, CEACAM5 levels also decreased, indicating that the stability of these proteins may be mutually interdependent (Fig. 5*C*). The induction of CEACAM5/6 expression by acidosis and acidosis plus hypoxia was partially reversible in SW1222 cells after 72 h recovery in pH 7.4 and 21% oxygen (*SI Appendix*, Fig. S6*A*). Using immunofluorescence staining of CEACAM5 and CEACAM6 in SW1222 cells, we observed that only a small subset of cells was CEACAM5- or CEACAM6-positive under physiological pHe and normoxic conditions (Fig. 5*D*). However, when expression was induced by either an acidic or a hypoxic environment, we observed a higher proportion of CEACAM5-positive/CEACAM6-positive cells and a greater degree of overlap between the two proteins. We observed a similar increase in CEACAM5/6 levels after acidity or hypoxia treatment in C99 and JHCOLOY1 cells (*SI Appendix*, Fig. S6 *B* and *C*).

### CEACAM5 and CEACAM6 Induction Is Independent of HIF1α, CDX1, or CDX2.

Given the strong induction of CEACAM5 and CEACAM6 observed in a subset of cell lines under hypoxic conditions, we tested whether this response was dependent on HIF1α. Arguing against this was that siRNA-mediated knockdown of HIF1α only mildly affected CEACAM5/6 protein levels (Fig. 5*E*). Since CDX1 was previously shown to control lumen formation in CRC cell lines (40, 42, 43), we hypothesized that CDX1 and CDX2 transcription factors might be required for CEACAM5/6 mRNA transcription. Furthermore, we found CDX1 and CDX2 to be enriched in an analysis of putative transcription factors for genes that correlated with acid resistance (*SI Appendix*, Table S3). However, siRNA-mediated reduction of CDX1 and/or CDX2 did not lead to a decrease of CEACAM6 protein in SW1222 cells. Surprisingly, knockdown of the two transcription factors led to a further increase of CEACAM6 in SW1222 (Fig. 5*F*) and C99 cells (*SI Appendix*, Fig. S6*D*). Taken together, these results indicate that CEACAM6 is involved in an acid- or hypoxia-triggered stress response in colorectal cancer cells but is not linked to HIF1α signaling or under control by CDX1/2. We next tested whether the induction of CEACAM5 and CEACAM6 under acidity and hypoxia was specific to these chemical properties, or whether that may be part of a more general stress response. Since acidity inhibits mTOR signaling (44), we tested whether pharmacologically inhibiting this pathway phenocopied the acid induction of CEACAM5/CEACAM6. However, rapamycin did not lead to a dose-dependent increase of CEACAM5/6 (*SI Appendix*, Fig. S6*E*).

### Lack of CEACAM6 Reduces Levels of pH-regulating Membrane Proteins.

**Fig. 6.**
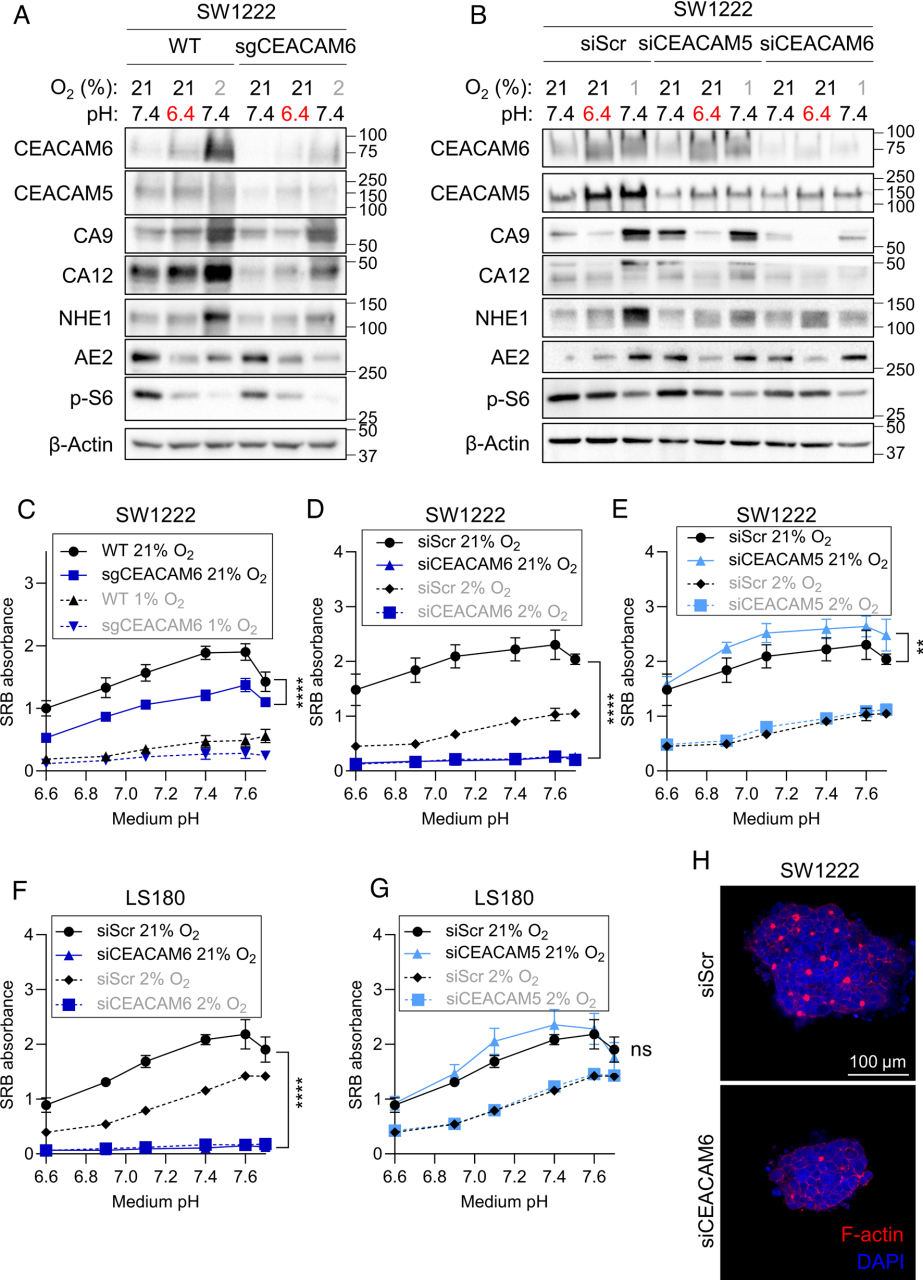
CEACAM6 is required for cell growth and expression of pH-regulating proteins. (*A*) Western blot of lysates from SW1222 WT and cells transduced with lentivirus for CRISPR/Cas9-mediated partial knockout of CEACAM6. Cells were treated with media of pHe 7.4 or pHe 6.4 under 21 or 2% O_2_. (*B*) Western blot of lysates from SW1222 cells treated with siScr, siCEACAM5, or siCEACAM6, followed by treatment with media of pHe 7.4 or pHe 6.4 under 21 or 1% O_2_. (*C*) Cell growth (SRB absorbance) at 6 d as a function of pHe in SW1222 WT cells transduced with lentivirus for CRISPR/Cas9-mediated partial knockout of CEACAM6 under 21 or 1% O_2_. Mean ± SEM of four independent repeats (triplicate technical replicates). (*D*) Cell growth (SRB absorbance) at 6 d as a function of pHe in SW1222 cells treated with siScr or siCEACAM6 under 21 or 2% O_2_. Mean ± SEM of three independent repeats (triplicate technical replicates). (*E*) Cell growth (SRB absorbance) at 6 d as a function of pHe in SW1222 cells treated with siScr or siCEACAM5 under 21 or 2% O_2_. Mean ± SEM of three independent repeats (triplicate technical replicates). (*F*) Cell growth at 6 d as a function of pHe in LS180 cells treated with siScr or siCEACAM6 under 21 or 2% O_2_. Mean ± SEM of three independent repeats (triplicate technical replicates). (*G*) Cell growth at 6 d as a function of pHe in LS180 cells treated with siScr or siCEACAM5 under 21 or 2% O_2_ conditions. Mean ± SEM of three independent repeats (triplicate technical replicates). (*H*) Images of single-cell colonies grown in Matrigel of SW1222 cells transfected with siScr or siCEACAM6 24 h prior to seeding. Colonies were stained with DAPI and TRITC-phalloidin (for F-actin labeling). Cells were cultured in a layer of Matrigel covered with medium of pHe 7.4 for 10 to 14 d. Images are representative of two independent repeats, carried out in technical duplicate.

We next tested for possible role of CEACAM5/6 in pHi regulation and cell growth by means of gene ablation. For this, we established a clonal cell line where CEACAM6 was reduced, but not fully knocked-out, using virally transduced sgRNAs and Cas9. sgCEACAM6-treated SW1222 cells had reduced CEACAM5 levels, indicating that this isoform’s stability requires the presence of CEACAM6 (Fig. 6*A*). We also observed modestly reduced levels of proteins implicated in pHi control in sgCEACAM6-treated cells, including CA9, CA12, and NHE1. In addition, the acid-loading membrane transporter AE2 was reduced. Interestingly, sgCEACAM6 cells showed a mild reduction in mTOR activity, indicated by a decrease in phospho-S6 (Ser240/244) levels siRNA-mediated knockdown of CEACAM6 resulted in a similar reduction of the pH-regulating proteins (CA9, CA12, NHE1, and AE2) as well as the mTOR marker phospho-S6. Given that inhibition of mTORC1 prevents HIF1α transcription (45), this could be the underlying cause of the reduction of CA9, CA12, and NHE1, which are all dependent on HIF1α. Our previous studies have demonstrated that AE2 degradation is also triggered by mTOR inhibition (5). Interestingly, siCEACAM5 did not show the same effect on levels of pH-regulating proteins, with unchanged levels of CA9, CA12, NHE1, and AE2 (Fig. 6*B*). Overall, our data indicate that CEACAM6 ablation has more pronounced consequences on pH regulation, compared to CEACAM5.

### CEACAM6, but Not CEACAM5, Is Necessary for Cell Growth.

Since acid-resistant cell lines have high CEACAM6 expression, we tested whether the presence of CEACAM6 confers a growth advantage, particularly at low pHe. We measured cell growth as a function of medium pH in sgCEACAM6- or siCEACAM6-treated cells under normoxic and hypoxic conditions (Fig. 6 *C* and *D*). A CRISPR/Cas9-mediated reduction of CEACAM6 led to slower cell growth, but the defect was more pronounced with siRNA-mediated knockdown. Strikingly, lack of CEACAM6 reduced growth across the pHe range, indicating that the growth-promoting effect of CEACAM6 is not unique to acidic conditions. Growth was greatly reduced under hypoxic conditions, and siCEACAM6 treatment led to its further decrease. In contrast to siCEACAM6, treatment with siCEACAM5 did not reduce cell growth (Fig. 6*E*). We observed a similar reduction in cell growth with siCEACAM6, but not siCEACAM5, in LS180 (Fig. 6 *F* and *G*) and JHCOLOY1 cells (*SI Appendix*, Fig. S7*A*). The lack of effect of siCEACAM5 on cell growth of JHCOLOY1 was confirmed using a second siRNA (*SI Appendix*, Fig. S7*B*). Next, we tested whether lack of CEACAM5 or CEACAM6 affects intracellular pH (pHi), reported using the pH-sensitive dye cSNARF-1 (*SI Appendix*, Fig. S6). Knockdown of CEACAM6 uniformly raised steady-state pHi in SW1222 cells (*SI Appendix*, Fig. S7*C*), which may relate to the decrease in AE2 levels in siCEACAM6-treated cells, i.e., reduced acid loading. Surprisingly, siCEACAM5 treatment had the opposite effect on resting pHi. We observed a similar, symmetrical effect of siCEACAM5/6 treatment on pHi in a second cell line, LS180 (*SI Appendix*, Fig. S7*D*). Finally, SW1222 cells treated with siCEACAM6 formed smaller colonies in Matrigel and showed impaired lumen formation (Fig. 6*H*), thus verifying a link between differentiation and CEACAM6.

### CEACAM5 and CEACAM6 Levels Are Raised in Human Colorectal Tumor Tissues.

**Fig. 7.**
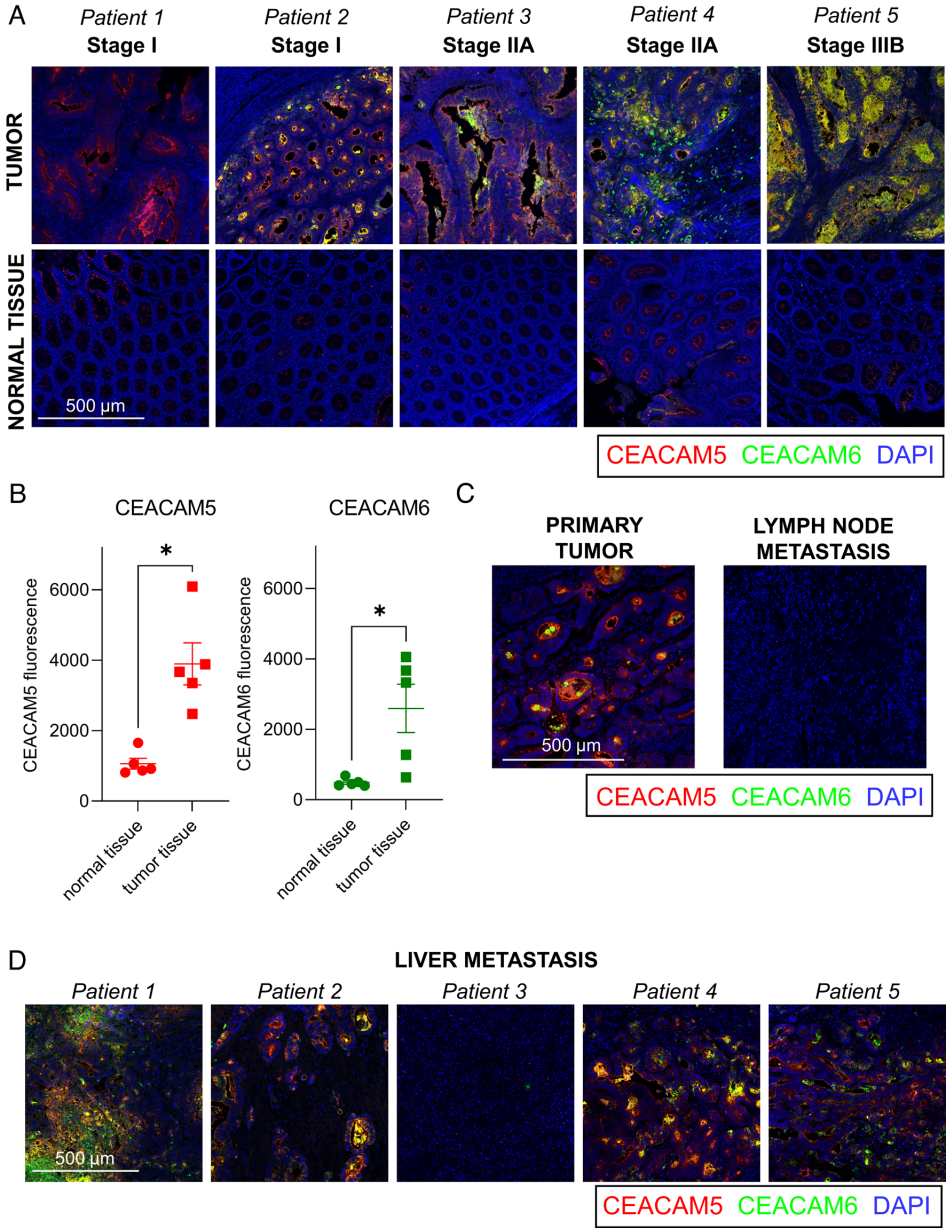
CEACAM6 is expressed in late-stage human colorectal tumor tissues. (*A*) Representative images of CEACAM5 (red), CEACAM6 (green), and DAPI (blue) fluorescence in matched pairs of human normal colon and tumor formalin-fixed and paraffin-embedded (FFPE) tissue sections from colorectal cancer patients. (*B*) CEACAM5 and CEACAM6 fluorescence intensity per cell in matched pairs of human normal and colon tumor tissues. Data are shown for five individual patients. Statistical significance was determined using paired two-tailed *t* test (*P < 0.05) (*C*) Representative images of CEACAM5 (red), CEACAM6 (green), and DAPI (blue) fluorescence in matched pairs of human primary tumor and lymph node metastatic tumor of formalin-fixed and paraffin-embedded (FFPE) tissue sections from colorectal cancer patients. (*D*) Representative images of CEACAM5 (red) and CEACAM6 (green) and DAPI (blue) fluorescence in human liver metastatic tumor FFPE) tissue sections from five colorectal cancer patients.

We first explored possible correlations between CEACAM5/CEACAM6 mRNA data and overall patient survival and disease-free patient survival using The Cancer Genome Atlas (TCGA) data. Without additional stratification, there was no emergent association between CEACAM5 or CEACAM6 levels and overall survival or disease-free survival among colon adenocarcinoma patients (*SI Appendix*, Fig. S8). For further insights, we measured patterns of CEACAM5 and CEACAM6 expression in CRC patient samples by stage of disease. While it is already known that CEACAM5 is highly expressed in multiple tumor types, with lower expression levels in primary epithelial cells (34, 46), the role of CEACAM6 is less established, although prognostic value has been demonstrated (37). In colorectal tumors matched with normal adjacent tissues, we observed a strong increase in both CEACAM5 and CEACAM6 levels in tumor tissues, compared to normal tissues (Fig. 7 *A* and *B*). However, CEACAM5 and CEACAM6 were present at low levels in normal adjacent tissues, in line with previously published single-cell RNA-seq data (46). Some tumor areas showed overlap between CEACAM5 and CEACAM6, but this was not always the case. CEACAM6 levels were very low in stage I tumor samples, but increased markedly with later tumor stages. We therefore hypothesize that CEACAM6 is a marker for late-stage cancers that are more likely acidic or hypoxic.

Both CEACAM5 and CEACAM6 have previously been implicated in promoting metastasis (32). CEACAM5-positive cancer cells have been reported in liver metastasis patient samples (47), and colorectal cell lines producing high levels of serum CEACAM5 were highly metastatic to the liver in athymic mice (48). Interestingly, CEACAM5 and CEACAM6 were absent in metastatic lymph node tumor tissue, compared to a matched primary tumor sample (Fig. 7*C*). but highly expressed in the majority of analyzed samples from human liver metastasis (Fig. 7*D*, with additional representative images shown in *SI Appendix*, Fig. S9).

## Discussion

Our phenotypic screen of a large panel of CRC cell lines identified CEACAM5 and CEACAM6 as highly expressed in acid-resistant phenotypes. We found that CEACAM5 and CEACAM6 are strongly induced by acidic conditions, with further induction under hypoxia, at least in some cell lines. We hypothesize that CEACAM6 induction is a response to acid stress and confers cancer cells with a growth advantage under such conditions. This contrasts with CEACAM5, which is not required for cell survival. Furthermore, we show that CEACAM6 is highly expressed in samples from late-stage tumor patients.

We postulate that CEACAM6 could be used as a biomarker of aggressive tumors that are associated with acidity. An association between high levels of CEACAM6 and survival was previously reported in PDAC patients (49). CEACAM6’s surface localization and low expression in normal tissues makes it a promising antigen for therapies targeting acidic tumor regions, particularly if these are also hypoxic. This strategy may be particularly beneficial to improve the efficacy of radiotherapy or conventional chemotherapies, which tend to be less effective in underperfused regions. For example, bispecific antibodies could be developed for directing T cells specifically to poorly perfused regions of the tumor microenvironment, with limited accessibility for immunotherapies. Alternatively, antibody–drug conjugates (ADCs) targeting CEACAM6 as the cancer-specific antigen and cytotoxic drugs as the cargo, could be developed. Given the link between OXPHOS inhibition and pH sensitivity (27), using ADCs with OXPHOS inhibitors, such as atovaquone as cargo and specific targeting to acidic regions marked by CEACAM6 may be a promising strategy. This approach would alleviate concerns about off-target reactions of OXPHOS inhibitors and allow for an increased dosage where it is needed. Finally, other drug delivery platforms, such as exosomes, may provide an attractive way to deliver cytotoxic cargo specifically to hard-to-treat regions of aggressive tumors. Given CEACAM6’s important role in promoting cell growth, inactivating its function using siRNAs (50) or inhibitory antibodies (32, 51) without any additional cargo also deserves further research.

Due to the prominent role of CEA as a blood biomarker, further research is needed to understand the extent to which CEACAM6 is also shed into the bloodstream (52). Efficiency of CEACAM6-targeted therapies may be compromised if these chelate with blood-borne CEACAM6 fragments. Furthermore, distribution in healthy tissues needs to be considered, and the consequences of CEACAM6 inhibition in nontumor tissues must be understood. Our data suggest that targeting CEACAM6 may be ineffective in metastatic tumors of the lymph nodes and early-stage tumors; therefore, other therapies may be more appropriate in these instances.

In summary, we identified CEACAM6 as a biomarker for acid-resistant clones in colorectal cancer, induced further by acidity, and highly expressed in later-stage disease. Targeting CEACAM6, either by inhibiting its function or using it as a means for directing other therapeutic payloads to the tumor microenvironment, is a promising research avenue.

## Materials and Methods

### Cell Lines and Culture Conditions.

The study used human colorectal cell lines from commercial or academic sources that divide outside the body, have been deidentified, and are not relevant material under the Human Tissues Act (UK). Cell lines were cultivated in DMEM (Gibco 41965-039), supplemented with 10% FBS (Sigma-Aldrich) and 1% PS (100 U mL^−1^ penicillin and 100 µg mL^−1^ streptomycin; Sigma-Aldrich). For subsequent cell growth and metabolic acid production experiments, cells were treated with medium based on NaHCO_3_-free DMEM (Sigma-Aldrich, Cat. No. D7777), supplemented with various concentrations of NaHCO_3_ and NaCl. Medium pH was set by adjusting [HCO_3_^−^], achieved by mixing various ratios of stocks containing either 44 mM NaHCO_3_ (pH 7.7 at 5% CO_2_) or 44 mM NaCl (pH 6.2 at 5% CO_2_). This protocol maintains constant medium osmolarity. Cells were maintained at 37 °C and 5% CO_2_. Cell lines were authenticated by single nucleotide polymorphism (SNP)-based profiling and tested routinely for mycoplasma contamination.

### Cell Growth Analysis using SRB.

Cells were plated in triplicate at densities of 4,000 cells/well in clear, flat bottom 96-well plates (Costar). The following day, the medium was replaced with 200 µL medium of different pHe as indicated in figure legends. Cells were cultured for 6 d, and extracellular pH was monitored on each day using phenol red absorbance. After 6 d, the cells were fixed using 10% trichloroacetic acid (TCA) at 4 °C for 60 min. Afterward, they were washed with H_2_O four times and stained with SRB (0.057% in 1% acetic acid) for 30 min. Washing four times with 1% acetic acid removed residual SRB. The remaining SRB was dissolved in Tris base (10 mM) and its absorbance read at 520 nm (Cytation 5 imaging plate reader; BioTek).

### Competition Assays.

Cells were transduced with lentivirus containing either pLV eGFP (Addgene plasmid #36083) or pLV mCherry2 (Addgene plasmid #36084) constructs in a six-well plate (Costar) using a multiplicity of infection (MOI) of 10. After 48 h following transduction, the virus was removed, and the cells were washed with PBS. Cells were seeded in a black, flat bottom 96-well plate (Ibidi) at 8,000 cells per well (4,000 cells of eGFP-labeled cells and 4000 cells of mCherry2-labeled cells). The following day, the medium was replaced with 400 µL medium of different pH as indicated in figure legends. The cells were cultivated for 9 d, and the medium was replaced every 3 d. After 9 d, the medium was replaced with PBS, and the cells were imaged using the Cytation 5 imaging plate reader. Images of fluorescence excited at 377 nm and collected at 447 nm (eGFP), and of fluorescence excited at 531 nm and collected at 640 nm (mCherry2), were acquired using a 10× objective. Using Gen5 software (BioTek), we determined the area covered by either eGFP or mCherry2 fluorescence to calculate fractional abundance.

### Analysis of pH-related Phenotypes and Their Gene Expression Correlates.

For 68 CRC cell lines, cell growth after 6 d of treatment with a range of medium pHs was measured using the SRB assay. In parallel, medium pH was assessed daily using phenol red absorbance measurements. Experiments were carried out in triplicate, and two to fifteen biological repeats were carried out for each cell line. For each cell line, curves (least squares to biphasic fitting) were fitted based on an average of multiple biological repeats to determine the optimal medium pH (pH max) as well as the pH which leads to a 50% reduction in cell growth (pH_50_). Principal component analysis (PCA) was carried out on normalized cell growth data as a function of pHe (at 6 pHe values ranging from pHe 6.6 to pHe 7.7) to identify similar phenotypes within the cell line panel. PCA was also carried out based on cumulative acid production. Gaussian mixture modeling, a statistical approach previously described by our group (38), was used to classify cell lines into distinct groups using pH_50_ values and metabolic flux data was used to classify cell lines into distinct groups: acid-sensitive, intermediate and acid-resistant for cell growth data, and low metabolic acid production, intermediate, and high metabolic acid production for cumulative acid production data. Expression data were obtained from a linked publication available at https://github.com/jeffliu6068/GMMchi. For the detection of differentially expressed genes, multiple *t* tests were carried out by comparing lines assigned to the extreme groups. All data analysis workflows were carried out using MATLAB.

### Animals.

Xenografts were established according to a modification of a previously published protocol (27). Subcutaneous injections of CRC cells were performed on 12-wk athymic Nude Crl:NU(NCr)-Foxn1nu female mice. Briefly, SW1222 or COLO320HSR cells were resuspended in 100 mL of a 1:1 mixture of Matrigel and serum-free DMEM. Two million SW1222 cells were injected on the left flank and two million COLO320HSR cells on the right flank. Animals were then randomly allocated to drink water (N = 6) or bicarbonate water (N = 6). Mice were weighed, and tumor dimensions were measured three times a week. At the end of the experiments (humane end point), mice were injected with Var3 pH-(low)-insertion peptide (pHLIP) fluorescently labeled with Cy5.5 (0.7 nmol/g in sterile PBS) and Hoechst 33342 (10 mg/kg in sterile PBS). After allowing 20 min for circulation, mice were killed, and tumors were excised for histology. Animal procedures were carried out in accordance with national and institutional guidelines, ethics and welfare board instructions, and under the authority of Home Office Project License PPL P01A04016.

See *SI Appendix* for further methods.

## Supplementary Material

Appendix 01 (PDF)

## Data Availability

All study data are included in the article and/or *SI Appendix*. Previously published data were used for this work (38).
