# Supplementary material for: Phenotypic screen of sixty-eight colorectal cancer cell lines identifies CEACAM6 and CEACAM5 as markers of acid resistance
Source: Proc Natl Acad Sci U S A. 2024 Mar 19;121(13):e2319055121. doi: 10.1073/pnas.2319055121 (PMC10990159; doi:10.1073/pnas.2319055121)
Supplement: Supplementary file 1 — Appendix 01 (PDF) [file pnas.2319055121.sapp.pdf]

PNAS

Supporting Information for

Phenotypic screen of sixty-eight colorectal cancer cell lines identifies CEACAM6 and CEACAM5 as markers for acid-resistance

Johanna Michl[a], Bobby White[a,1], Stefania Monterisi[a,1], Walter F. Bodmer[b,2], Pawel Swietach[a,2]

[a] Department of Physiology, Anatomy and Genetics, Parks Road, Oxford OX1 3PT, UK
[b] MRC Weatherall Institute for Molecular Medicine, John Radcliffe Hospital, Headington, Oxford OX3 9DS, UK
[1]B.W. and S.M. contributed equally to this work.
[2]To Whom correspondence may be addressed: Walter Bodmer and (walter.bodmer@hertford.ox.ac.uk) and Pawel Swietach (pawel.swietach@dpag.ox.ac.uk)

This PDF file includes:

Supporting text
Figures S1 to S9
Tables S1 to S4
SI References

Supporting Materials and Methods

Monitoring medium pH using absorbance. Medium pH was measured by phenol red absorbance at 430 and 560 nm using Cytation 5 imaging plate reader (Biotek) equipped with a CO_2 gas controller (Biotek). Measurements were taken from 200 µL medium in clear, flat-bottom 96-well plates (Costar) with lids at 37 °C and 5% CO_2. Media were based on $NaHCO_3$-free DMEM (Sigma-Aldrich, Cat. No. D7777), supplemented with 10% FBS, 1% PS and various concentrations of $NaHCO_3$ and NaCl, as indicated in figure legends. Phenol red absorbance ratios were converted to pH values using a calibration curve of HEPES/MES-supplemented media at eight different pH values recorded at 0% CO_2.

Immunoblotting. Samples were prepared by lysing the cells using radioimmunoprecipitation assay (RIPA) buffer containing proteinase and phosphatase inhibitors. Protein concentration in the samples was measured using bicinchoninic acid (BCA) protein assay kit and adjusted using water. Samples were not heated, and loaded onto a 10% acrylamide gel. The gel was run at 90 V for 15 minutes and at 120 V for 90 minutes. Afterwards, membrane transfer was performed at 90 V for 90-120 minutes. Primary antibodies against CEACAM5 (Invitrogen 14-0669-82), CEACAM6 (Invitrogen MA5-29144), CDX1 (Abcam ab126748), CDX2 (Cell Signalling D11D10), AE2 (Novus Biologicals NBP2-15301), CA9 (a gift from Prof. Silvia Pastoreková, Slovak Academy of Sciences), CA12 (Novus Biologicals NBP2-46042), NHE1 (BD #611774), HIF-1α (BD #610958), phospho-S6 (Ser240/244, Cell Signalling #5364P) or β-Actin (Proteintech HRP-60008) were applied overnight. Afterwards, membranes were incubated in either and goat anti-rabbit (Invitrogen A32731) or anti-mouse secondary antibody (Invitrogen A32727) (1:5000), and the membrane was visualized using horseradish peroxidase. Antibody binding of β-Actin protein was used as a loading control.

siRNA transfections. Cells were plated at 200 000 cells/well in a clear, flat bottom 6-well plate in 3 mL DMEM supplemented with 10% FBS and 1% PS. 10 nM Dharmacon smartpool siRNA (siScr, siCEACAM5, siCEACAM6, siCDX1, siCDX2, siHIF1α) was resuspended in 300 mL optiMEM. In a separate tube, 6 mL Lipofectamine RNAiMAX transfection reagent were mixed with 300 mL optiMEM. siRNA and transfection reagent mixtures were combined and incubated at room temperature for 10 min. Afterwards, the mixture was added to cells in suspension and incubated for 48 h. Experiments were performed 72 h after siRNA-transfection

qPCR. Total cellular RNA was isolated (RNeasy kit, Qiagen, 74104) from cells cultured for 48 h hours in sodium bicarbonate/CO_2 buffered conditions of either pH 7.4 or pH 6.4 in 6-well plates. iScript Advanced cDNA synthesis Kit was used for the reversed transcription reaction into cDNA. The resulting cDNA was diluted 1:10 in DNAse-free water before quantification by real-time PCR. qPCR was carried out using Taqman probes for CEACAM6 and β-Actin. 2x Taqman Fast Advanced Mastermix was used for PCR reactions at standard cycling conditions using Applied Biosystems StepOne System. The transcript levels were normalized with the β-Actin transcript level and data were represented as fold change relative to the average of control samples. Data are representative of three independent experiments.

Immunofluorescence. For immunofluorescence experiments on cultured cells, cells were cultured in Ibidi 12-well slides as indicated in figure legends. After three days, cells were fixed with 4% paraformaldehyde for 10 minutes at RT. After 10 min of permeabilization with 0.2% Triton X-100, 1 h of blocking with 3% BSA, cells were incubated with primary antibodies for 1.5 – 24 h (Anti-CEACAM5 (Invitrogen 14-0669-82), 1:400, anti-CEACAM6 (Invitrogen MA5-29144), 1:400) followed by secondary antibodies for 1 h (anti-mouse Alexafluor 555, anti-rabbit Alexaflour 488, 1:500) and staining with Hoechst 33342 for 1 min. Slides were mounted with Antifade Gold mounting medium. Images were acquired using a 40x objective on the Zeiss LSM 700 confocal microscope.

CRISPR/Cas9-mediated gene knock-out. LentiCRISPR v2 was a gift from Feng Zhang (Addgene plasmid # 52961; http://n2t.net/addgene:52961; RRID:Addgene_52961). sgRNA sequences were cloned into LentiCRISPR v.2 backbone using the manufacture's protocol. Briefly, LentiCRISPR v 2 backbone was digested using BsmBI enzyme for 1 h at 37 °C. After gel purification, the linearized DNA was ligated with oligo duplex using the Quick Ligation Kit (NEB M2200S). Plasmid DNA was transformed into DH5alpha competent cells (NEB C2987H). The sgRNA sequence used for CEACAM6 was GGACATGCAATCTGCAGGGA. Virus aliquots were prepared by the Virus Production Facility at WIMM, University of Oxford. SW1222 cells were plated in clear, flat-bottom 6-well plate at a density of 200,000 cells/well and transduced using a 500 mL aliquot of lentivirus carrying the LentiCRISPR v2 construct encoding for sgRNA sequences targeting *CEACAM6.*Polybrene was added at a concentration of 4 µg/mL. The 6-well plate was incubated for two days before puromycin (5 µg/mL) was added for selection, and cells were incubated for three days. Infected cells were seeded at 2 cells/well in 200 µL of media in wells of 96-well plates. Single-cell clones were established and tested for CEACAM6 protein levels using Western blotting.

Intracellular pH measurements. pH was measured using cSNARF1 in cells identified by particle analysis of fluorescence centered around nuclei visualized using the DNA-binding stain Hoechst-33342. Cells were plated in triplicate at 50,000-100,000 cells per well in black wall, flat coverslip bottom Ibidi μ-plate 96-well plates with a growth area of 0.56 cm 2 per well (Ibidi) and were left to attach overnight. They were then incubated in media supplemented with cSNARF1-AM (5 mg/mL, Invitrogen) and the nuclear stain Hoechst-33342 (10 mg/mL, Molecular Probes), for 15 min, and then replaced with medium of varying sodium bicarbonate concentration (twice). Images of fluorescence excited at 377 nm and collected at 447 nm (Hoechst-33342), and of fluorescence excited at 531 nm and collected at 590 nm and 640 nm (cSNARF1), were acquired using Cytation 5 imaging plate reader (Biotek) and its bespoke software. Images were acquired using a 10x objective. Measurements were performed in an atmosphere of 37° C and 5% CO_2, established in the plate reader. Further analysis of the population distribution of pH data was performed using a MATLAB script. cSNARF1 fluorescence ratios were converted into pHi using a calibration curve obtained through the nigericin method. pHi distributions from replicate wells were pooled, and low intensity measurements were removed using a second MATLAB script.

Gene Expression Analysis. Total RNA from cells was extracted using the RNeasy kit (Qiagen) according to the manufacturer's instructions. All samples were processed in accordance with the Affymetrix protocol, and 2 μg of fragmented and labeled cDNA was hybridized to the Affymetrix GeneChip U133+2 arrays.

Lumen formation assay. To determine the differentiation potential of individual cell lines, lumen formation assays were carried out according to a previously published protocol (40). Briefly, single-cell suspensions were achieved by filtration through 30 μm MACS smart strainers (Miltenyi). Five hundred cells were suspended in 50 μL of an ice-cold mixture of Matrigel and diluted 1:1 with ice-cold DMEM medium of varying sodium bicarbonate concentration (44-2.75 mM sodium bicarbonate). Cell suspensions were seeded in duplicate into 96-well plates precoated with a solidified 1:1 Matrigel/DMEM layer (30 μL per well). Overlaid Matrigel was allowed to set at 37° C, and medium of varying pHe was then added to the wells. Colonies were grown for 10-14 days, with medium changes every 3 days. The medium was removed and 100 μL of 4% (v/v) paraformaldehyde in PBS was added for 20 minutes. Fluid was then removed and 100 mL of PBS containing 1:200 dilution of Triton-x was added for 10 minutes. The wells were washed 4 times with 50 mmol/L glycine in PBS. 100 μL TRITC-phalloidin diluted at 1:1,000 in PBS was added and incubated at 4° C overnight. The colonies were then rinsed 3 times with PBS and a total of 100 μL of 10 μg/mL 406-diamidino-2-phenylindole (DAPI; Sigma) in PBS was added to each well. The plate was imaged using a Zeiss LSM 700 confocal microscope (Carl Zeiss Ltd.).

Histology of human tissue sections and mouse xenograft tumors. Matched pairs of tumor and normal colon formalin fixed paraffin embedded human tissue (FFPE) sections were obtained from BioChain. Samples were de-identified from donors and, as a commercially-provided tissue section, fall outside the Human Tissues Act (UK). The sections were heated for 60 °C for 1 h and de-waxed using xylene for 3 x 5 min. After rehydration (5 min in 100/90/70% ethanol), slides were washed 3 times in dH_2O. After permeabilization in 0.1% triton-X100 for 10 min, blocking was performed in 10% FBS in PBS-Tween (0.1%) for 1 h. Sections were incubated with primary antibodies against CEACAM5 (Invitrogen 14-0669-82) and CEACAM6 (Invitrogen MA5-29144)overnight (1:200 in 3% BSA in PBS-Tween). After incubation with secondary antibodies for 1 h (1:500, anti-rabbit Alexafluor 555, anti-mouse Alexa-fluor 488) and staining with 300 nM DAPI for 10 min, slides were mounted with Antifade Gold mounting medium. Images were acquired using a 10x objective on the Zeiss LSM 700 confocal microscope. Tissues from mouse xenografts were cryo-sectioned. Fresh-frozen sections were mounted with Drop-n-stain mounting medium (containing DAPI). Images were acquired using a 10x objective on a Leica confocal microscope.

Table S1. Replication error (RER), epithelial-mesenchymal transition (EMT) and mutation status of driver mutations in colorectal cancer (*PIK3CA, p53, KRAS, BRAF, APC, CTNNB1, FBXW7, TGFBR2* and *SMAD4*). Data originally described in Liu *et al.* (1). 1 = negative, 2= positive, NA = data not available. For acid-sensitivity clusters, 1 = acid-sensitive, 2 = intermediate, 3 = acid-resistant.

Cell lines	Acid-sensitivity Cluster	RER status	*APC*	*p53*	*CTNNB1*	*KRAS*	*BRAF*	*PIK3CA*	*FBXW7*	*TGFBR2*	*SMAD4*	EMT status
C10	2	1	1	2	1	1	1	1	1	1	1	1
C106	2	1	2	2	1	2	1	1	1	1	1	1
C2BBE1	2	1	2	2	2	1	1	1	1	1	2	1
C32	3	1	2	2	1	1	1	1	2	1	2	1
C99	3	1	1	1	1	1	1	1	1	1	1	1
CACO2	3	1	2	2	2	1	1	1	1	1	2	1
CAR1	1	1	1	2	1	1	1	1	NA	NA	1	1
CC20	2	1	NA	2	1	1	1	1	NA	NA	NA	2
CCK81	2	2	2	2	2	1	2	2	2	2	1	1
CCO7	2	1	2	2	1	2	1	1	2	NA	NA	2
CL40	2	NA	2	2	NA	2	1	2	1	1	1	1
COLO205	2	1	2	2	2	1	2	1	1	1	1	1
COLO206	1	1	2	2	2	1	2	1	1	1	1	1
COLO320DM	1	1	2	2	1	1	1	1	1	1	1	2
COLO320HSR	1	1	2	2	1	1	1	1	1	1	1	2
COLO678	2	1	2	1	1	2	1	1	1	1	1	1
CX1	2	1	2	2	1	1	2	2	1	2	2	1
DLD1	2	2	2	2	1	2	1	2	2	2	1	1
GP2D	2	2	2	1	2	2	2	2	2	2	1	1
GP5D	2	2	2	1	2	2	2	2	2	2	1	1
HCC2998	2	1	2	2	1	2	1	2	2	1	2	1
HCC56	3	NA	NA	2	NA	2	1	1	1	1	1	1
HCT116	2	2	1	1	2	2	1	2	1	2	1	1
HCT15	2	2	2	2	1	2	1	2	2	2	1	1
HDC111	3	1	NA	NA	NA	2	1	2	NA	NA	2	1
HDC114	2	1	2	2	NA	1	1	2	NA	NA	NA	1
HDC54	2	1	2	2	2	1	1	1	NA	NA	NA	1
HDC57	2	1	2	2	2	1	1	1	NA	NA	NA	1
HDC82	2	1	2	2	1	1	1	1	1	1	1	1
HDC9	3	2	2	2	NA	1	1	2	NA	NA	NA	1
HRA19	2	1	2	2	1	1	1	2	1	1	1	1
HT29	3	1	2	2	1	1	2	2	1	2	2	1
HT55	2	1	2	2	2	1	2	1	2	1	1	1
ISCEROL	1	NA	NA	NA	NA	NA	NA	NA	NA	NA	NA	1
JHCOLOY1	3	NA	NA	NA	NA	NA	NA	NA	NA	NA	NA	1
LOVO	2	2	2	1	1	2	1	1	2	2	1	1
LS174T	3	2	1	1	2	2	2	2	1	2	1	1
LS180	3	2	1	1	2	2	2	2	1	2	1	1
LS411	3	2	2	2	1	1	2	1	2	2	1	1
LS513	3	1	1	1	2	2	2	1	1	1	1	1
NCIH508	2	1	1	2	1	1	2	2	1	1	1	1
NCIH548	1	1	2	2	2	1	2	1	1	1	1	1
NCIH747	3	1	2	2	1	2	1	1	1	1	1	1

Table S1 (continued). Replication error (RER), epithelial-mesenchymal transition (EMT) and mutation status of driver mutations in colorectal cancer (*PIK3CA, p53, KRAS, BRAF, APC, CTNNB1, FBXW7, TGFBR2* and *SMAD4*). Data originally described in Liu *et al.* (1). 1 = negative, 2= positive, NA = data not available. For acid-sensitivity clusters, 1 = acid-sensitive, 2 = intermediate, 3 = acid-resistant.

Cell lines	Acid-sensitivity Cluster	RER status	*APC*	*p53*	*CTNNB1*	*KRAS*	*BRAF*	*PIK3CA*	*FBXW7*	*TGFBR2*	*SMAD4*	EMT status
OUMS23	3	NA	2	2	NA	1	2	1	1	1	1	1
OXCO1	2	1	2	2	NA	NA	2	1	NA	NA	NA	2
OXCO2	2	2	2	NA	NA	NA	1	1	2	NA	NA	1
PMFKO14	3	NA	1	2	2	NA	NA	NA	NA	2	NA	1
RCM1	3	1	2	2	1	2	1	1	2	1	2	1
RKO	2	2	2	1	1	1	2	2	1	2	1	1
RW2982	3	1	2	1	1	2	1	1	1	1	2	1
RW7213	3	1	2	1	1	2	1	1	1	1	1	1
SKCO1	3	1	2	1	1	2	1	1	1	1	1	1
SNU1235	2	1	NA	NA	NA	NA	NA	NA	NA	NA	NA	1
SNUC1	3	1	1	2	1	1	1	NA	1	2	1	1
SNUC2B	3	2	1	2	1	2	1	NA	2	2	1	1
SW1222	3	1	2	2	1	2	1	NA	1	1	2	1
SW1417	3	1	2	2	1	1	2	NA	1	1	1	1
SW403	3	1	2	2	1	2	2	NA	1	1	1	1
SW48	2	2	2	1	2	1	1	NA	2	2	1	1
SW480	2	1	2	2	1	2	1	NA	1	1	1	2
SW620	3	1	2	2	1	2	1	NA	1	1	1	2
SW837	2	1	2	2	1	2	1	NA	2	1	1	1
SW948	2	1	2	2	1	2	1	NA	1	1	2	1
T84	2	1	2	NA	1	2	1	NA	1	1	2	1
VACO10MS	3	1	2	2	1	2	1	NA	2	1	2	1
VACO4A	3	1	2	2	1	2	1	NA	NA	NA	2	1
VACO4S	2	1	2	2	1	2	1	NA	NA	NA	2	1
VACO5	3	2	2	2	1	2	2	2	NA	NA	NA	1

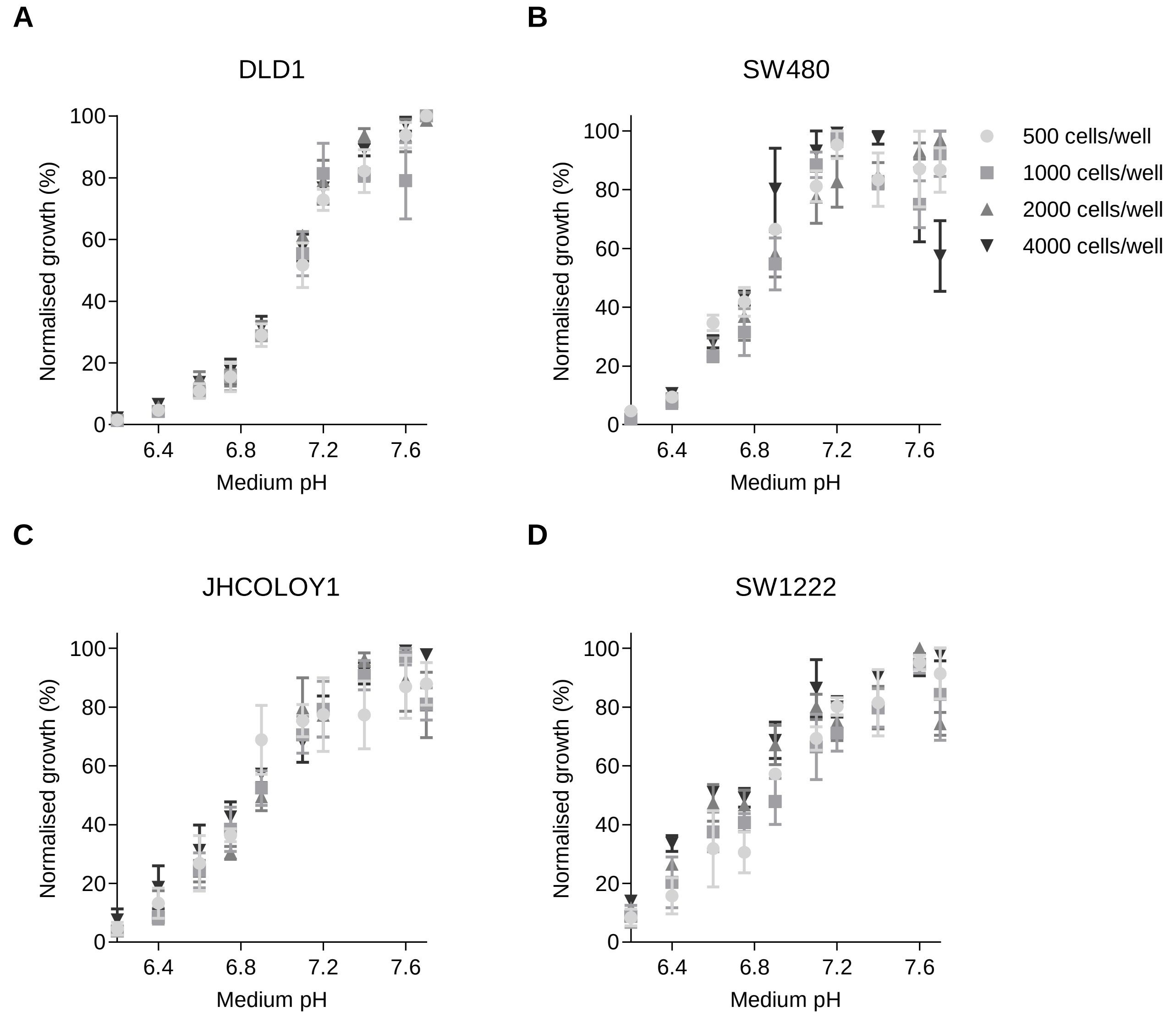

Fig. S1. (A-D) Normalised cell growth after 6 days of culture from a range of starting pHe. SRB absorbance values were normalized to growth at optimum pHe. Cell lines were seeded at densities of 500, 1000, 2000 or 4000 cells/well. Mean ± SEM of two to five independent repeats (triplicate technical replicates).

A

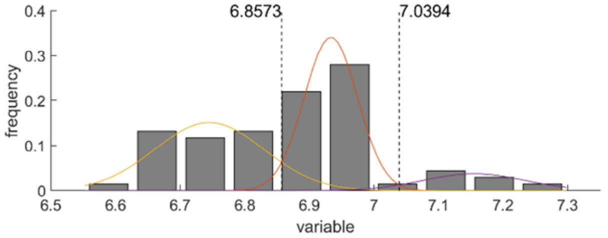

B

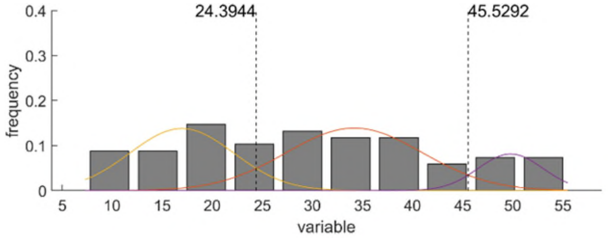

C

	Acid-sensitive	Inter-mediate	Acid-resistant
CEACAM5 low	6	14	4
CEACAM5 high	0	20	24

$p = 0.0002$

D

	Acid-sensitive	Inter-mediate	Acid-resistant
CEACAM6 low	6	16	6
CEACAM6 high	0	18	22

$p = 0.0007$

Fig. S2. (A) Gaussian mixture modelling of frequency distributions of pH_{50} and (B) metabolic flux at pHe 7.7, plotted as the x-axis variable. Best fit shows three Gaussian curves, the sum of which produces the best description of the distribution by Akaike Information Criterion (AIC). (C) Correlation between CEACAM5, or (D) CEACAM6 mRNA expression levels and acid-sensitivity phenotype. Cell lines were classified as low or high for CEACAM5/6 mRNA expression based on Gaussian mixture modelling analysis. Significance determined using Fisher's exact test from 2x3 tables shown.

Table S2. Contingency analysis for correlation between acid-sensitivity phenotype and replication error (RER), epithelial-mesenchymal transition (EMT) and mutation status of several driver mutations in colorectal cancer. We used Fisher's exact test (based on 2x3 tables shown) to test for correlation with RER, EMT, *PIK3CA, p53, KRAS, BRAF, APC, CTNNB1, FBXW7, TGFBR2* and *SMAD4* mutation status.

RER status	acid-sensitive	intermediate	acid-resistant	
positive	0	10	6	p = 0.456
negative	5	23	18	

EMT	acid-sensitive	intermediate	acid-resistant	
positive	2	4	1	p = 0.079
negative	4	30	27	

PIK3CA	acid-sensitive	intermediate	acid-resistant	
positive	0	13	6	p = 0.118
negative	5	14	12	

p53	acid-sensitive	intermediate	acid-resistant	
positive	5	24	19	p = 0.661
negative	0	7	7	

KRAS	acid-sensitive	intermediate	acid-resistant	
positive	0	16	17	**p = 0.026***
negative	5	15	9	

BRAF	acid-sensitive	intermediate	acid-resistant	
positive	2	9	9	p = 0.711
negative	3	24	17	

APC	acid-sensitive	intermediate	acid-resistant	
positive	4	29	18	p = 0.153
negative	1	3	7	

CTNNB1	acid-sensitive	intermediate	acid-resistant	
positive	2	10	5	p = 0.562
negative	3	19	18	

FBXW7	acid-sensitive	intermediate	acid-resistant	
positive	0	12	5	p = 0.097
negative	4	15	17	

TGFBR2	acid-sensitive	intermediate	acid-resistant	
positive	0	10	7	p = 0.324
negative	4	15	16	

SMAD4	acid-sensitive	intermediate	acid-resistant	
positive	0	6	9	p = 0.234
negative	5	20	15	

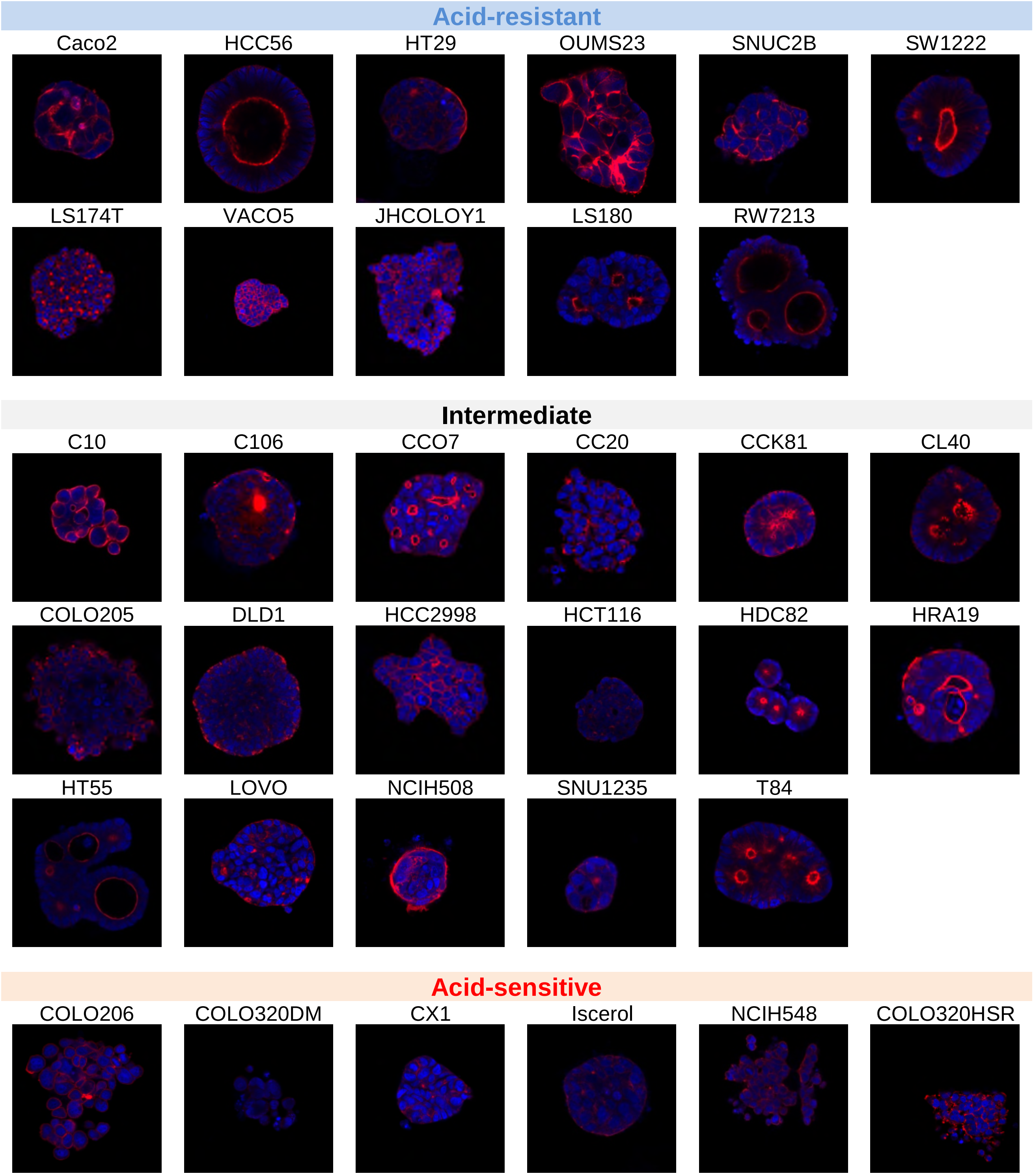

Fig. S3. Representative images of single cell colonies grown in Matrigel at pHe 7.4, stained with TRITC-phalloidin and DAPI.

Table S3. Contingency table analysis to seek correlation between lumen formation phenotype and either CEACAM5 or CEACAM6 mRNA expression levels. Cutoffs between low and high expression were determined using Gaussian mixture modelling (GMM). Significance determined using Fisher's exact test based on 2x3 tables.

Lumen formation:	**negative**	**intermediate**	**positive**
CEACAM5 low	9	4	0
CEACAM5 high	2	6	13

p < 0.0001**

Lumen formation:	**negative**	**intermediate**	**positive**
CEACAM6 low	10	5	0
CEACAM6 high	1	5	13

p < 0.0001**

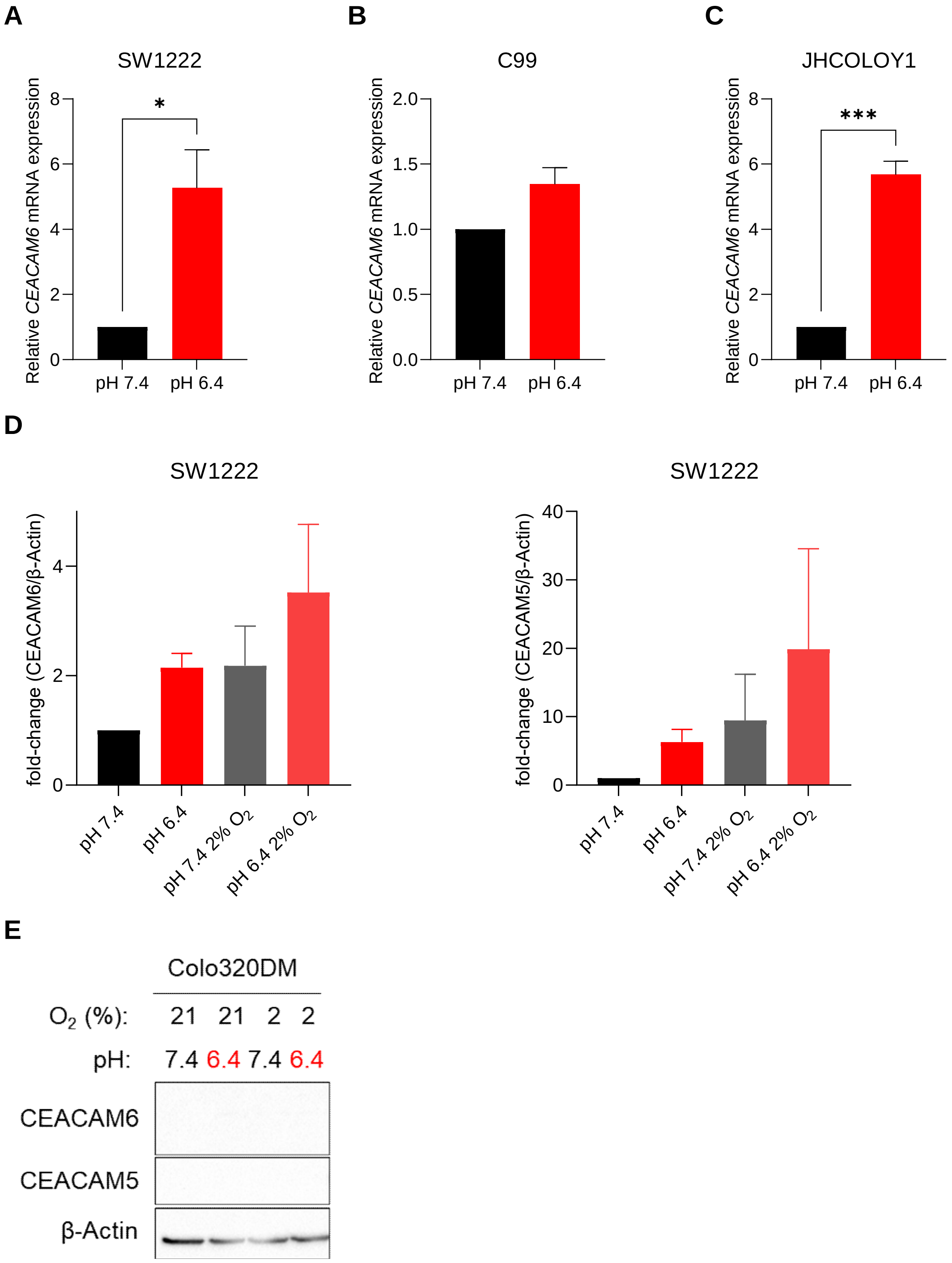

Fig. S4. (A-C) fold-change mRNA expression levels of CEACAM6 after 48 h of treatment with media of pH 7.4 or pH 6.4 in SW1222, C99 and JHCOLOY1 cells. (D) Quantification of western blot data shown in Figure 5C. Quantification of signal intensities, relative to β.-Actin. Mean ± SEM of 3-5 independent repeats. (E) Western blot of lysates from COLO320DM cells after treatment for 72 h with media of pHe 7.4 or pHe 6.4 under 21% or 2% O_2 conditions.

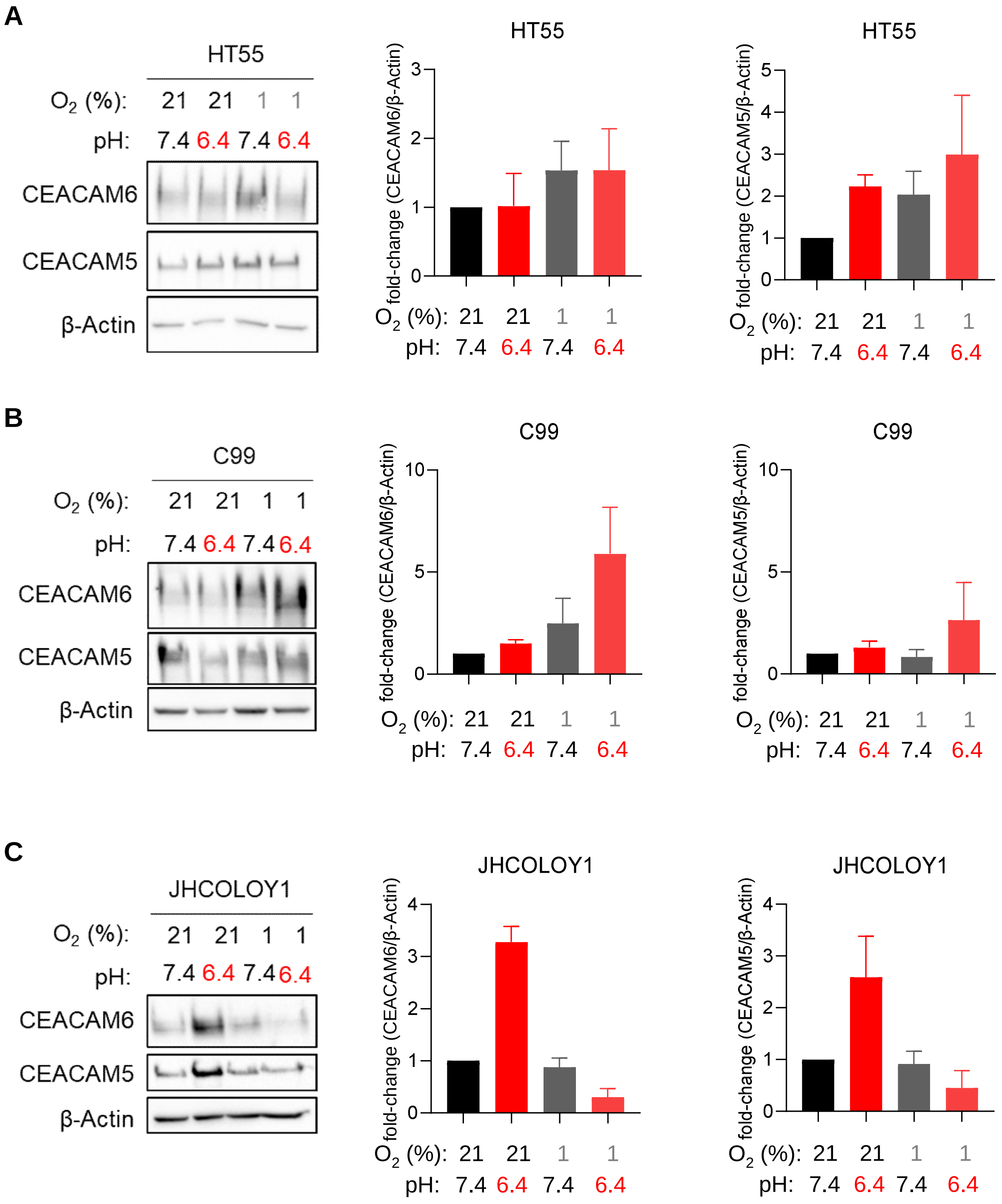

Fig. S5. (A-C) Quantification of western blot data from HT55, C99 and JHCOLOY1 cells after treatment for 72 h with media under indicated conditions. Quantification of signal intensities relative to β.-Actin. Mean ± SEM of three independent repeats.

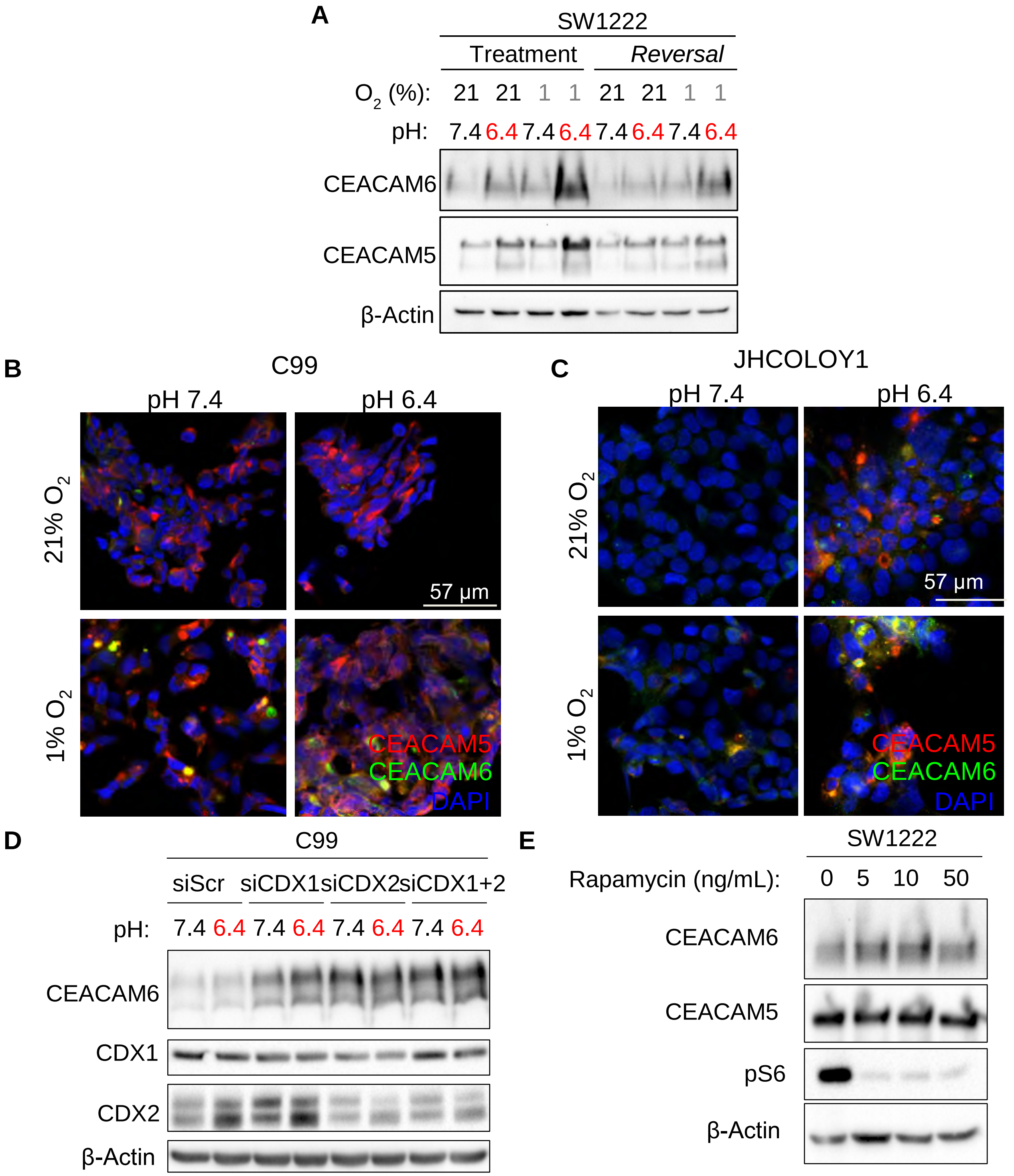

Fig. S6. (A) Western blot of lysates from SW1222 cells treated for 72 h with media of pHe 7.4 or 6.4 under either 21% or 1% O_2 conditions ("treament"). After a period of treatment, cells were returned to pHe 7.4 medium under 21% O_2 conditions for another 72 h ("reversal"). (B) Immunofluorescence staining of C99 and (C) JHCOLOY1 cells after treatment for 72 h with media of pHe 7.4 or 6.4 under either 21% or 1% O_2 conditions. (D) Western blot of lysates from SW1222 cells transfected with siScr, siCDX1, siCDX2 or siCDX1+2, followed by treatment for 48 h with media of pHe 7.4 or 6.4. (E) Western blot of lysates from SW1222 cells after treatment with 0, 5, 10 or 50 ng/mL rapamycin for 72 h.

Table S4. Transcription factor enrichment analysis for bimodally distributed genes which are significantly higher expressed in acid-resistant cell lines. Analysis based on previously annotated transcription targets assembled from multiple resources, using ChEA3 (2). CEACAM5 and CEACAM6 are highlighted in bold. Genes with known transcription factor function are highlighted in red.

Transcription factor	Score	Overlapping Genes
ISX	2	GUCY2C, VAV3, CNTNAP2, DDC, GPX2, CDX1, REG4, IHH, ATP10B, TM4SF5, LRRC31, HEPH, MEP1A, MYO1A, **CEACAM5**, AGR3, CDHR5, TRIM31, TINAG, NOX1, FOXA3
CDX2	3.4	GUCY2C, DDC, PKDCC, WWC2, IHH, WNK4, ATP10B, PLD1, ETS2, ZFP36L2, LRRC31, HEPH, AGR3, TINAG, GPX2, CDX1, REG4, PRLR, MEP1A, MYO1A, **CEACAM6**, **CEACAM5**, CDHR5, TRIM31, NOX1, FOXA3
CDX1	7.667	GUCY2C, DDC, GPX2, REG4, IHH, ATP10B, ETS2, LRRC31, HEPH, MEP1A, MYO1A, **CEACAM5**, AGR3, CDHR5, TRIM31, TINAG, NOX1, FOXA3
HNF4A	9.833	GUCY2C, DDC, WWC2, IHH, WNK4, PTPRM, CLDN2, ETS2, ZFP36L2, LRRC31, ABCC6P1, HEPH, BLNK, GPX2, CDX1, REG4, TM4SF5, PRLR, MEP1A, MYO1A, **CEACAM5**, NAPEPLD, NOSTRIN, CDHR5, WDFY3, TRIM31, FOXA3, NOX1
MYRFL	13	GUCY2C, DDC, HEPH, MEP1A, CDX1, MYO1A, REG4, IHH, AGR3, CDHR5, TRIM31, TINAG
HNF1A	16.33	GPX2, DDC, CDX1, IHH, TM4SF5, CLDN2, ABCC6P1, MEP1A, MYO1A, BLNK, CDHR5, TEAD1, FOXA3
HNF4G	21.2	GUCY2C, DDC, CDX1, REG4, WWC2, IHH, WNK4, ATP10B, TM4SF5, ZFP36L2, PAPSS2, LRRC31, HEPH, MEP1A, MYO1A, AGR3, CDHR5, TRIM31, TINAG, FOXA3
HNF1B	25	GPX2, DDC, WWC2, IHH, CLDN2, PLD1, TINAG, TEAD1, ETS2, FOXA3, LRRC31
NR1I2	29.75	GUCY2C, CHRM3, DDC, CDX1, REG4, IHH, TM4SF5, ETS2, PAPSS2, ABCC6P1, HEPH, MEP1A, MYO1A, CDHR5, FOXA3, BCL2L14
FOXA2	30.83	CA12, SAMD5, VAV3, CHRM3, GPX2, DDC, PKDCC, WWC2, CADPS, WNK4, PTPRM, TM4SF5, PLD1, ETS2, ZFP36L2, HS6ST2, ABCC6P1, MEP1A, TEAD1, FOXA3
CREB3L3	39.33	GUCY2C, DDC, CDX1, REG4, TM4SF5, CLDN2, ABCC6P1, HEPH, MEP1A, MYO1A, NOSTRIN, CDHR5, FOXA3
GLI3	59.67	CNTNAP2, TIMP2, PTPRM, SLC1A3, TEAD1, ZFP36L2, HS6ST2
NR1H4	61.67	ABCC6P1, DDC, MEP1A, MYO1A, TM4SF5, CDHR5, CLDN2, PAPSS2, FOXA3
FOXA3	67	GUCY2C, GPX2, DDC, PKDCC, CDX1, IHH, TM4SF5, ETS2, PAPSS2, ABCC6P1, HEPH, MYO1A, CDHR5
JUN	69.5	VAV3, CHRM3, CNTNAP2, CDX1, WWC2, PTPRM, SLC1A3, PLD1, ZFP36L2, ETS2, PAPSS2, HS6ST2, MEP1A, NOSTRIN, TIMP2, WDFY3, TEAD1
TRPS1	72.33	VAV3, SLC4A7, CA12, AGR3, WDFY3, TEAD1, PRLR, ZFP36L2, LRRC31
NKX23	72.5	SLC4A7, GUCY2C, CHRM3, HEPH, MEP1A, CDX1, MYO1A, CDHR5, ETS2, ZFP36L2
CREB3L2	77	WWC2, TIMP2, PTPRM, TEAD1, ZFP36L2, PAPSS2
PGR	82.5	CA12, VAV3, CNTNAP2, TIMP2, AGR3, PRLR, PAPSS2
ZEB1	82.8	SLC4A7, GPX2, TIMP2, SLC1A3, WDFY3, TEAD1, ETS2, ZFP36L2, FOXA3

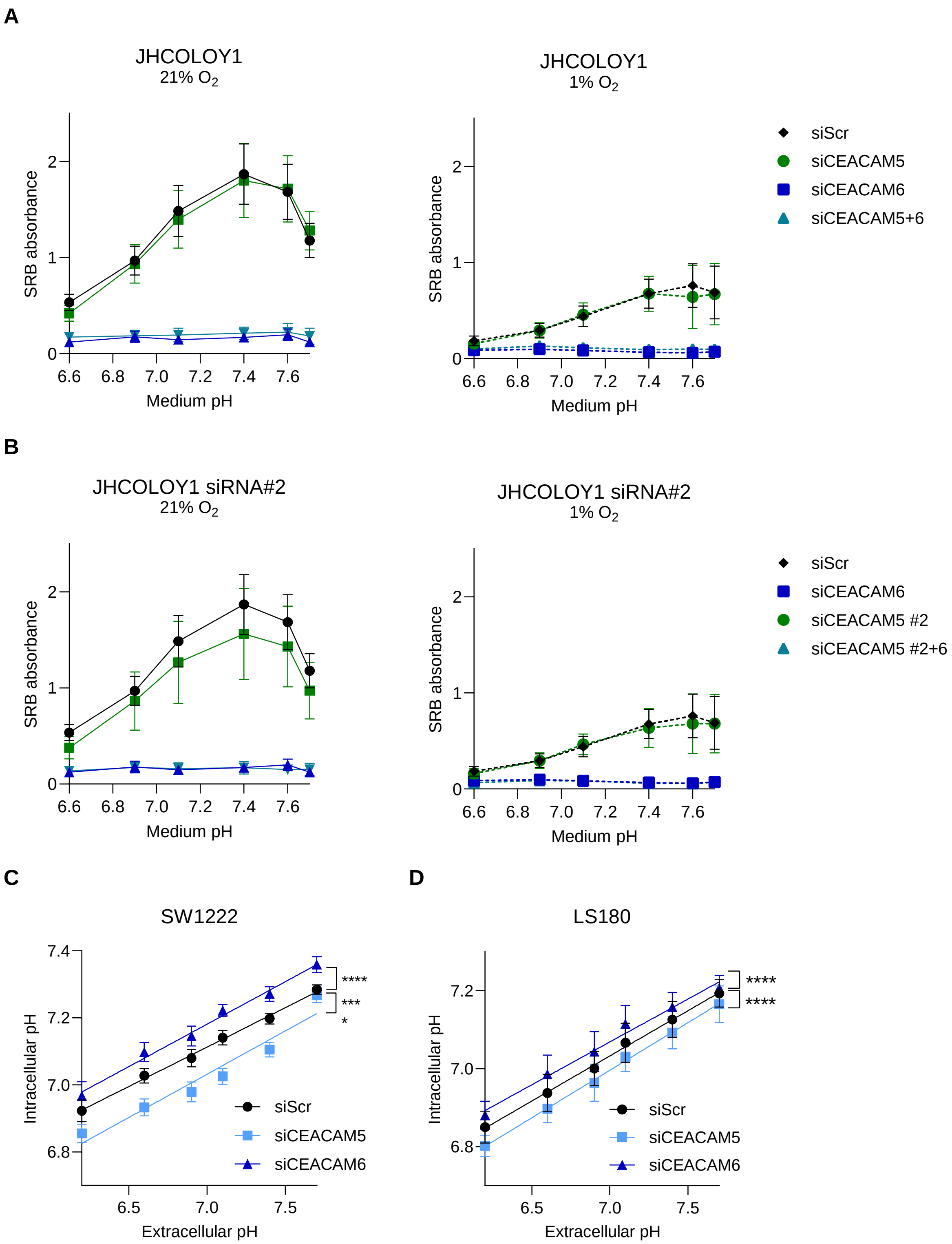

Fig. S7. (A-B) Cell growth (sulforhodamine B [SRB] absorbance) at 6 days as a function of pHe in JHCOLOY1 cells treated with siScr, siCEACAM5 (A) or siCEACAM5#2 (B), siCEACAM6 or siCEACAM5+6 under 21% or 2% O_2 conditions. Mean ± SEM of three independent repeats (triplicate technical replicates). (B) (C-D) pHi-pHe relationship of SW1222 and LS180 cells treated with siScr, siCEACAM5 or siCEACAM6 96 h prior to measurement.

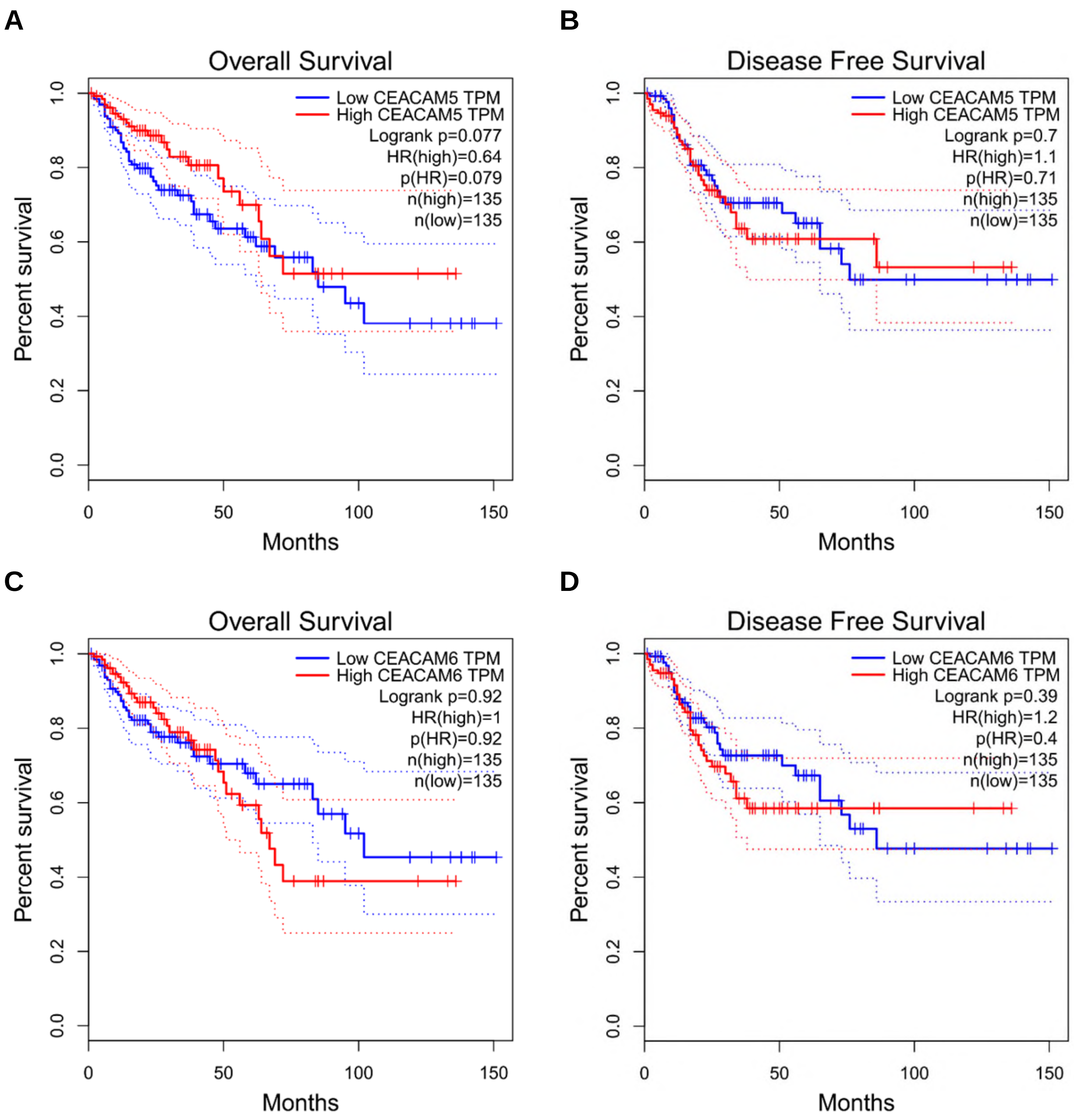

Fig. S8. Correlation between overall survival/disease free survival and CEACAM5 (A-B) mRNA levels and CEACAM6 mRNA levels (C-D). Analysis was performed using the GEPIA tool (3), based on TCGA and GTEx data sets.

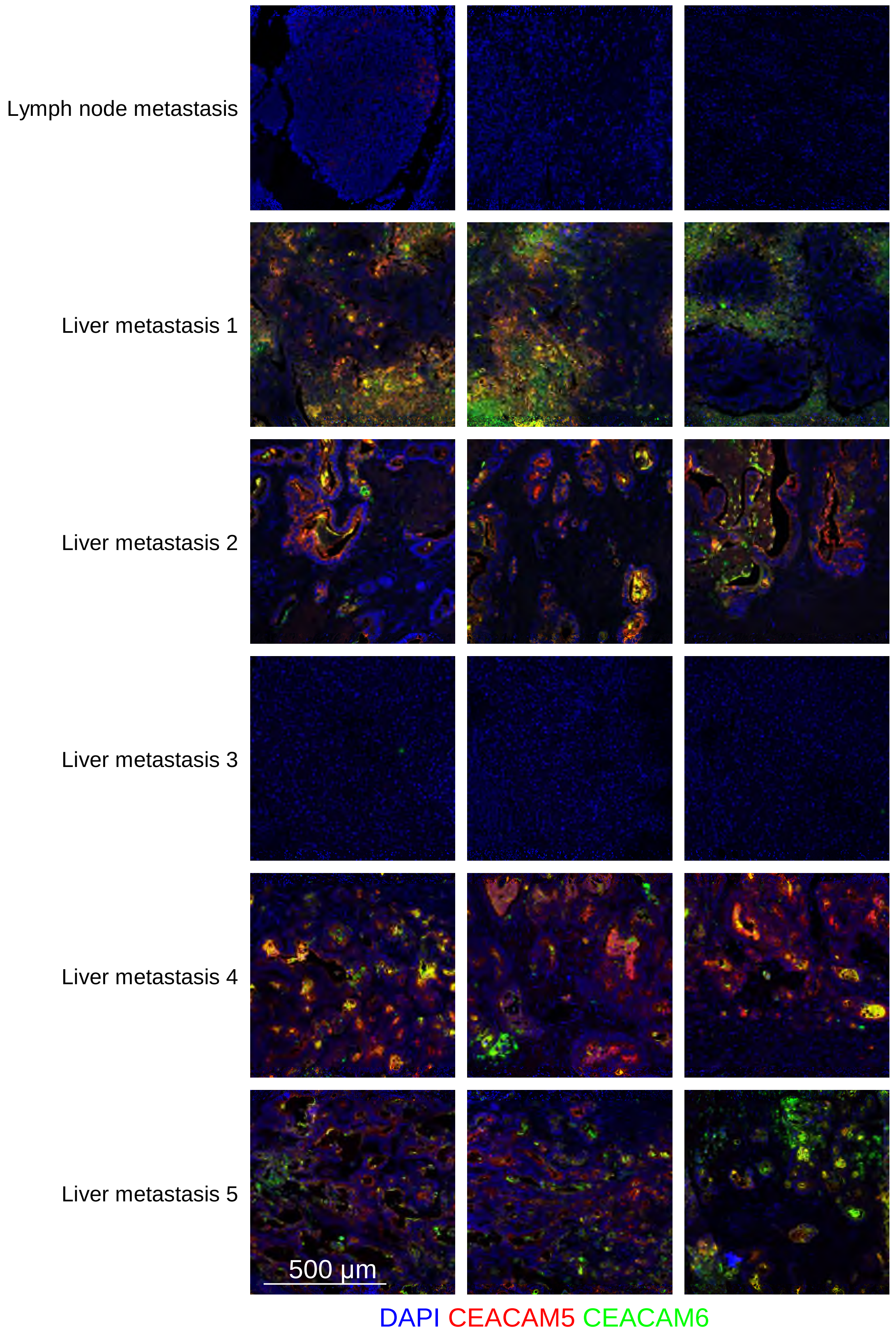

Fig. 9. Representative images of FFPE-sections from human metastatic tumours from either lymph nodes or liver metastasis stained with and CEACAM6 (green), CEACAM5 (red) and DAPI.

SI References

1. T.-C. Liu, P. N. Kalugin, J. L. Wilding, W. F. Bodmer, GMMchi: Gene Expression Clustering Using Gaussian Mixture Modeling. *bioRxiv*, 2022.2002.2014.480329 (2022).
2. A. B. Keenan *et al.*, ChEA3: transcription factor enrichment analysis by orthogonal omics integration. *Nucleic Acids Res* **47**, W212-w224 (2019).
3. Z. Tang, C. Li, B. Kang, G. Gao, Z. Zhang, GEPIA: a web server for cancer and normal gene expression profiling and interactive analyses. *Nucleic Acids Res* **45**, W98-w102 (2017).
